# Mechanism of agonist-induced activation of the human itch receptor MRGPRX1

**DOI:** 10.1371/journal.pbio.3001975

**Published:** 2023-06-22

**Authors:** Bing Gan, Leiye Yu, Haifeng Yang, Haizhan Jiao, Bin Pang, Yian Chen, Chen Wang, Rui Lv, Hongli Hu, Zhijian Cao, Ruobing Ren

**Affiliations:** 1 Shanghai Key Laboratory of Metabolic Remodeling and Health, Institute of Metabolism and Integrative Biology, Fudan University, Shanghai, China; 2 The Kobilka Institute of Innovative Drug Discovery, School of Medicine, The Chinese University of Hong Kong, Shenzhen, Guangdong, China; 3 State Key Laboratory of Virology, College of Life Sciences, Wuhan University, Wuhan, China; 4 Shenzhen Research Institute, Wuhan University, Shenzhen, China; 5 Shanghai Qi Zhi Institute, Shanghai, China; University of Zurich, SWITZERLAND

## Abstract

Mas-related G-protein-coupled receptors X1-X4 (MRGPRX1-X4) are 4 primate-specific receptors that are recently reported to be responsible for many biological processes, including itch sensation, pain transmission, and inflammatory reactions. MRGPRX1 is the first identified human MRGPR, and its expression is restricted to primary sensory neurons. Due to its dual roles in itch and pain signaling pathways, MRGPRX1 has been regarded as a promising target for itch remission and pain inhibition. Here, we reported a cryo-electron microscopy (cryo-EM) structure of G_q_-coupled MRGPRX1 in complex with a synthetic agonist compound 16 in an active conformation at an overall resolution of 3.0 Å via a NanoBiT tethering strategy. Compound 16 is a new pain-relieving compound with high potency and selectivity to MRGPRX1 over other MRGPRXs and opioid receptor. MRGPRX1 was revealed to share common structural features of the G_q_-mediated receptor activation mechanism of MRGPRX family members, but the variable residues in orthosteric pocket of MRGPRX1 exhibit the unique agonist recognition pattern, potentially facilitating to design MRGPRX1-specific modulators. Together with receptor activation and itch behavior evaluation assays, our study provides a structural snapshot to modify therapeutic molecules for itch relieving and analgesia targeting MRGPRX1.

## Introduction

Itch is defined as the sensation that causes the desire to scratch the skin [1]. It is a common and frequently occurring symptom associated with many skin diseases among humans [2]. Numerous factors can induce itches, such as chemicals, insect bites, and even self-generated substances resulting from varied diseases [3]. Unfortunately, due to diverse inducements and complicated pathogenesis, treating itch in the clinic is still challenging, especially the chronic itch, which will devastate people and cause much suffering [4]. The itch can be generally divided into histaminergic and nonhistaminergic [5]. Usually, most histaminergic itch results in acute itch, whereas chronic itch is more probable to be nonhistaminergic [6]. Therefore, the well-developed antihistamine drugs are inefficient in chronic itch relieving, which suggests the significance of finding novel drug targets for chronic itch treatment [6].

Mas-related G-protein-coupled receptors (MRGPRs) have been recently identified as pruritogenic receptors mediating the nonhistaminergic itch [7]. The *Mrgpr* gene family encodes MRGPRs, a large family which comprises 27 and 8 members in mice and humans, respectively [7,8]. MRGPRX1-X4 are 4 primate-specific receptors, suggesting that the X subfamily may be a simplified alteration in human evolution [4]. MRGPRX1 is the first identified human MRGPR that expresses in dorsal root ganglia (DRG) and trigeminal ganglia (TG) specifically [4]. Compared with other MRGPRX members, MRGPRX1 stands out for its dual roles in mediating itch [9] and inhibiting persistent pain [10]. Persistent pain is a severe health problem worldwide, and ordinary analgesics like opioids targeting opioid receptors may lead to several side effects such as drug addiction [10,11]. Notably, MRGPRX1 is insensitive to the classical opioid receptor antagonists, indicating that MRGPRX1 could be a new target for treating chronic pain [10].

Extensive studies of MRGPRX1 were conducted in itch and pain sensations, and inflammation [7]. A series of natural and synthetic agonists, antagonists, and allosteric modulators of MRGPRX1 have been developed [12]. However, there is currently no drug targeting MRGPRX1 commercialized. The structure determination of GPCR may provide the detailed molecular basis of ligand interaction to facilitate modulator development [13]. Recently, 2 groups reported the agonist-stabilized cryo-electron microscopy (cryo-EM) structures of MRGPRX2 and MRGPRX4 in complexes with trimeric G proteins [14,15]. The structural characteristics of orthosteric pockets and modulator specificities are examined thoroughly. The critical acidic residues D184^5.38^ and E164^4.60^ in MRGPRX2 and the entirely positive orthosteric pocket in MRGPRX4 mainly determine the chemical property of varies modulators. The active structures of MRGPRX1 with varies of modulators are also reported [16].

In this study, we reported the cryo-EM structure of the active MRGPRX1-G_q_ complex bound to compound 16 at an overall resolution of 3.0 Å. Compound 16 is a new synthetic MRGPRX1 agonist with high potency and selectivity over other MRGPRXs and opioid receptor [17]. Our complex structure reveals the conserved mechanism of small molecule-induced receptor activation among MRGPRX receptors. The structure also clearly presents a highly conserved orthosteric pocket for natural agonist recognition, such as bovine adrenal medulla 8–22 peptide (BAM8-22) [18], γ2-MSH [19,20], and conopeptide (CNF-Tx2) [21]. Notably, a few variable residues in orthosteric pocket of MRGPRX1 exhibit the unique agonist recognition pattern for compound 16. These findings will give us clues to the modification of small molecule scaffolds targeting MRGPRX1 specifically, potentially accelerating the development of novel drugs for the modulation of itch and pain.

## Results

### The overall structure of G_q_-coupled MRGPRX1 bound to compound 16

To improve receptor expression, we fused thermostabilized apocytochrome b_562_ (BRIL) [22] at the N-terminus of MRGPRX1. NanoBit tethering strategy [23] was used for the complex formation, with the LgBit and HiBit fused to the C-terminus of the receptor and G_β_ subunit, respectively (S1A and S1B Fig). We used bioluminescence resonance energy transfer (BRET) assay to evaluate the impact of receptor modification on G protein coupling capability. The fusion of BRIL and LgBit to receptor only marginally affected receptor activity (S1C Fig and S1 Table). To further stabilize the complex, we used an engineered G_αq_ chimera in the complex assembly. The engineered G_αq_ chimera was designed based on the mini-G_αs/q71_ [24,25] with several modifications (S1D Fig). Briefly, the N-terminal 1–18 residues of the mini-G_αs/q71_ [24] were replaced by corresponding N-terminal sequences of the human G_αi1_, while the α-helical domain of G_αi1_ was subsequently inserted into the mini-G_αs/q71_, thus providing possible binding sites for 2 antibody fragments scFv16 and Fab-G50 [26,27]. Additionally, 2 dominant-negative mutations (G203A and A326S) were introduced to decrease the affinity of nucleotide binding [28]. The same engineered G_αq_ chimera had been successfully used in the structure determination of several G_q_-bound GPCRs, including the G_q_-bound ghrelin receptor [29] and bradykinin receptors [30]. The engineered G_αq_ chimera used in the further structure study will be simplified as G_αq_. We co-expressed BRIL-MRGPRX1-Lgbit, G_αq_, and G_βγ_-HiBit to obtain the MRGPRX1-G_αq_ complex. The complex was further stabilized by incubating with Nb35 [31] and scFv16 [32] in the presence of compound 16 (S2 Fig).

**Fig 1:**
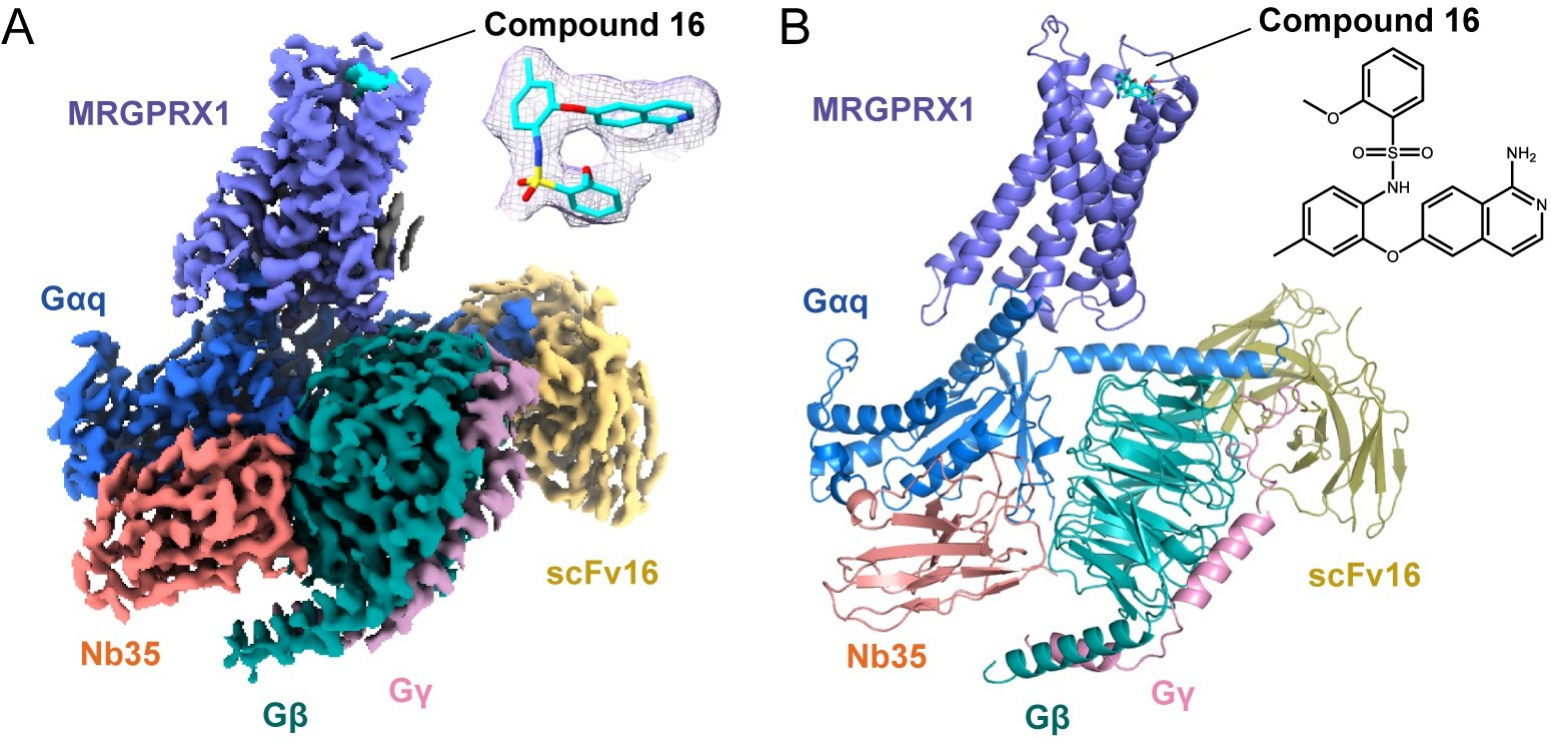
Cryo-EM structure of compound 16-MRGPRX1-G_αq_ complex. (A) Cryo-EM density map of MRGPRX1-G_αq_ in complex with compound 16. Receptor, compound 16, G_αq_, G_β_, G_γ_, Nb35, and scFv16 are colored slate, cyan, marine, forest, pink, salmon, and yellow-orange, respectively. The density of compound 16 is shown in mesh at the top-right corner. Compound 16 is fitted and shown as sticks. (B) Structural model for the compound 16-MRGPRX1-G_αq_ complex. Receptor, ligand, G_αq_, G_β_, G_γ_, Nb35, and scFv16 are colored the same as (A). Compound 16 is shown as sticks. The 2D chemical structure of compound 16 is shown in the top-right corner. cryo-EM, cryo-electron microscopy.

The compound 16-MRGPRX1-G_αq_ complex structure was determined by cryo-EM to yield a final map at an overall resolution of 3.0 Å (Figs 1A and S3A–S3F and S2 Table). In the map, the densities for the receptor, G_αq_, G_βγ_, Nb35, scFv16, and compound 16 could be well distinguished, and the interface residues between MRGPRX1 and G_αq_ (α5-helix) were clearly defined (S3G Fig). Thus, we built a reliable atomic model based on the well-traced α-helices and aromatic side chains (Fig 1B). Due to the flexibility, the N-terminus (M1-K25), part of the extracellular loops (I90) and long C-terminal residues (R279-Q322) of the receptor are invisible.

### The orthosteric pocket of MRGPRX1

**Fig 2:**
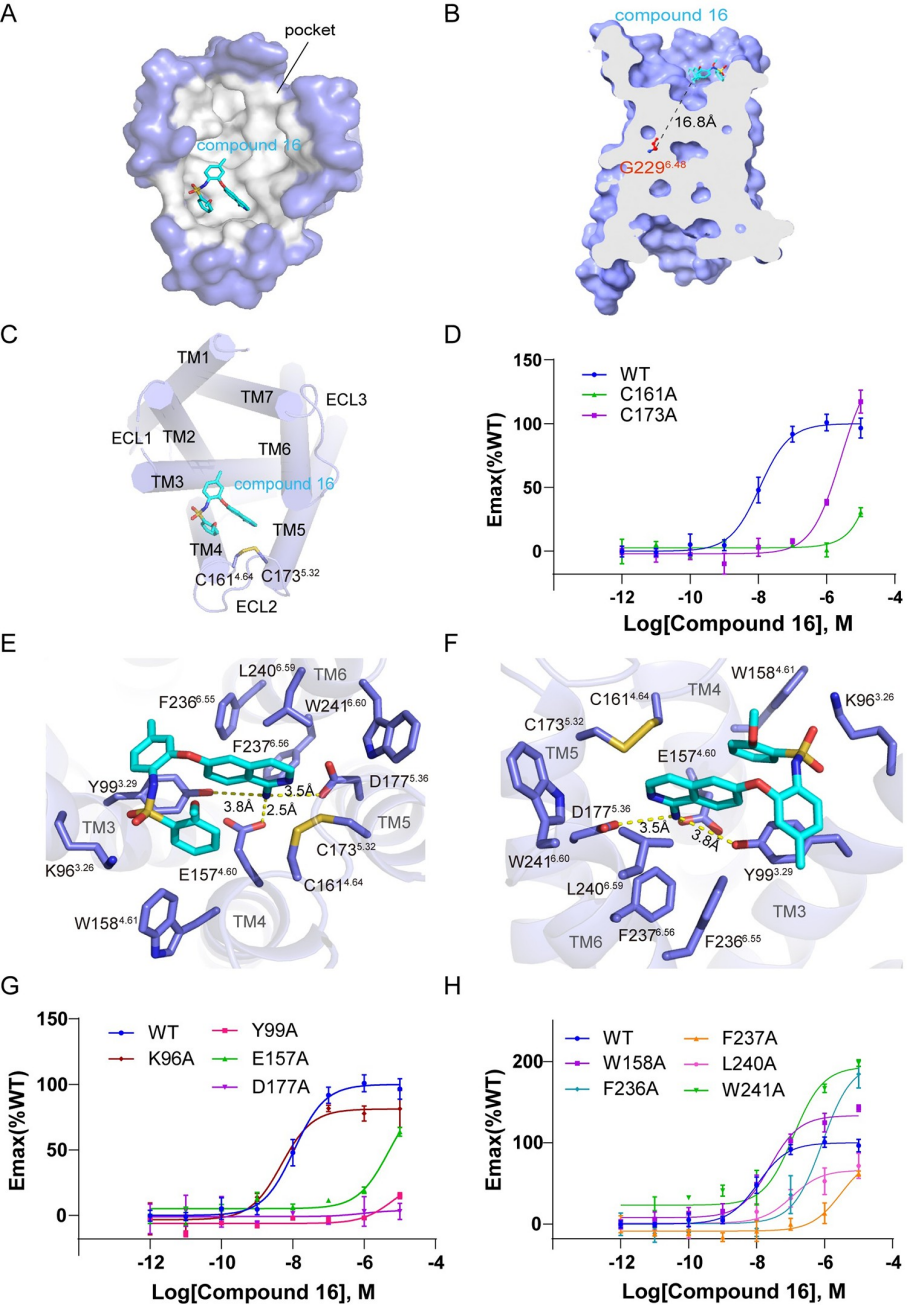
The binding pocket of compound 16 on MRGPRX1. (A) Top view of the compound 16-binding pocket from the extracellular side (surface mode). Pocket is colored gray, and compound 16 is shown as cyan sticks. (B) Cut-away view of the compound 16-binding pocket. The distance between the ligand and the traditional toggle switch position is shown as dashed lines. (C) Top view of the compound 16-binding pocket from the extracellular side (cartoon mode). TMs and ECLs are colored slate. Compound 16 is shown as cyan sticks. C161^4.64^ and C173^5.32^ are shown as sticks and colored slate. (D) BRET validation of residues C161^4.64^ and C173^5.32^. (E, F) Interaction between compound 16 and MRGPRX1 from 2 views. The key residues are shown as sticks and colored slate. The polar interactions are shown as yellow dashed lines. (G, H) BRET validation of residues in compound 16-binding pocket. Data are presented as mean ± SEM. *n* = 3; Emax, maximum effect; WT, wild type. The underlying data for Fig 2D, 2G and 2H can be found in S1 Data. BRET, bioluminescence resonance energy transfer.

The MRGPRX1 exhibits a shallow, broad, and wide-open ligand-binding pocket (Fig 2A). The distance between compound 16 and the critical toggle switch residue G229^6.48^ is about 16.8 Å (Fig 2B), indicating that compound 16 is positioned near the extracellular surface but not buried deep in the receptor. The shallow pockets are also observed in the MRGPRX2-G_αq_ [14,15] and MRGPRX4-G_αq_ complex [14], suggesting the common pocket features among all MRGPRX receptors. Compound 16 occupies only about one-third of the pocket (Fig 2A) but is sufficient for the receptor activation. Similarly, the agonist (R)-ZINC-3573 and MS47134 take up only a small part of the pocket in MRGPRX2 and MRGPRX4, respectively (S4A and S4B Fig). The structure alignment shows that 3 agonists bind to different regions of the orthosteric pocket in 3 receptors, indicating distinct recognition mechanisms (S4C Fig). The electrostatic potential of the MRGPRX1 pocket is partially negative, partially positive, and partially hydrophobic (S4D Fig). In contrast, the electrostatic potential of the MRGPRX2 pocket is partially negative (sub-pocket 1) and partially hydrophobic (sub-pocket 2), and the electrostatic potential of MRGPRX4 pocket is positive (S4E and S4F Fig). These results suggest that MRGPRXs may prefer agonist scaffolds with distinct electro-properties.

The orthosteric pocket of MRGPRX1 accommodating compound 16 is composed of residues majorly located on TM3/4/5/6 (Fig 2C). C161^4.64^ and C173^5.32^ form a disulfide bond, which is conserved among MRGPRXs [14] (S5 Fig). Moreover, alanine substitutions of C161^4.64^ and C173^5.32^ nearly abolish the G_q_ coupling activity (Fig 2D). Interestingly, MRGPRX1 lacks the canonical disulfide bond between TM3 and ECL2 in other class A family GPCRs [33]. Taken together, the disulfide bond substitution in MRGPRX1 may help to reorganize the extracellular loops and maintain the wide-open orthosteric pocket.

### The interaction between compound 16 and MRGPRX1

Compound 16 adopts a hairpin conformation in the pocket due to the intramolecular π-π interaction of the 1-aminoisoquinoline and phenylmethyl groups (Fig 1A). Notably, the amino group of 1-aminoisoquinoline forms strong salt bridges with E157^4.60^ and D177^5.36^ (Fig 2E and 2F), 2 conserved residues in all MRGPRXs (S5 Fig). Alanine substitutions of E157^4.60^ and D177^5.36^ dramatically affect receptor activation, suggesting that these 2 acidic residues play a crucial role in ligand recognition (Figs 2G and S6 Fig and S1 Table). Additionally, the variable residue Y99^3.29^ recognizes compound 16 through 2 types of interactions, the π-π interaction with the middle aromatic ring of compound 16 and polar interaction with the amino group of 1-aminoisoquinoline (Fig 2E and 2F). Alanine substitution of Y99^3.29^ nearly abolishes the G_q_ coupling activity (Figs 2G and S6 and S1 Table). Thus, the variable residue Y99^3.29^ is essential for recognizing compound 16. Besides, the positive charge residue K96^3.26^ in MRGPRX1 is conserved in MRGPRX4 but substituted by a serine in MRGPRX2 (S5 Fig). K96^3.26^ in MRGPRX1 is close to compound 16 (Fig 2E and 2F), but S103^3.26^ in MRGPRX2 is far away from (R)-ZINC-3573 (S7A Fig). However, the alanine substitution of K96^3.26^ slightly affects the receptor activation (Figs 2G and S6 and S1 Table), suggesting that K96^3.26^ does not directly participate in compound 16 recognition. In contrast, the K96^3.26^ in MRGPRX4 is critical for MS47134 recognition [14] (S7B Fig). The variation of pocket residues may partly explain the agonist selectivity among MRGPRXs [12]. Apart from these closer residues, the farther hydrophobic residues around compound 16, such as W158^4.61^, F236^6.55^, F237^6.56^, L240^6.59^, and W241^6.60^, also participate in the ligand-binding pocket formation (Fig 2E and 2F). These hydrophobic residues, except for F237^6.56^, exhibit indirect and relatively weak interactions with compound 16 (Figs 2H and S6 and S1 Table). Alanine mutation of F237^6.56^ shows a notable impact on compound 16-induced MRGPRX1 activation (Figs 2H and S6 and S1 Table), indicating a potentially crucial role of F237^6.56^ in ligand recognition or receptor activation. However, the alanine substitution of F236^6.55^ only partially affects the receptor activation, double confirmed by BRET assay and calcium imaging assay (Figs 2H and S6 and S1 Table). It suggests that F236^6.55^ may help maintain the ligand-binding pocket instead of directly interacting with the ligand. Besides, the activity of compound 16 is partially reduced by substituting L240^6.59^ with alanine in the BRET assay (Fig 2H and S1 Table). In contrast, it is not affected in the calcium imaging assay (S6 Fig). The discrepancy may indicate L240^6.59^ is less important for the compound 16-binding pocket. Conversely, the corresponding residue L247^6.59^ in MRGPRX2 was reported to be critical for its agonist (R)-ZINC-3573 binding (S7A Fig), and Y240 is located on ECL3 of MRGPRX4, which is also important for the recognition of the agonist MS47134 [14,15] (S7B Fig). The differences in hydrophobic residues surrounding the ligand pocket illustrate the preference of ligand recognition among MRGPRXs. Together, these detailed structural analyses of compound 16-binding pocket can provide important information for a better understanding of the ligand recognition mechanism in MRGPRX1.

### Activation of MRGPRX1

**Fig 3:**
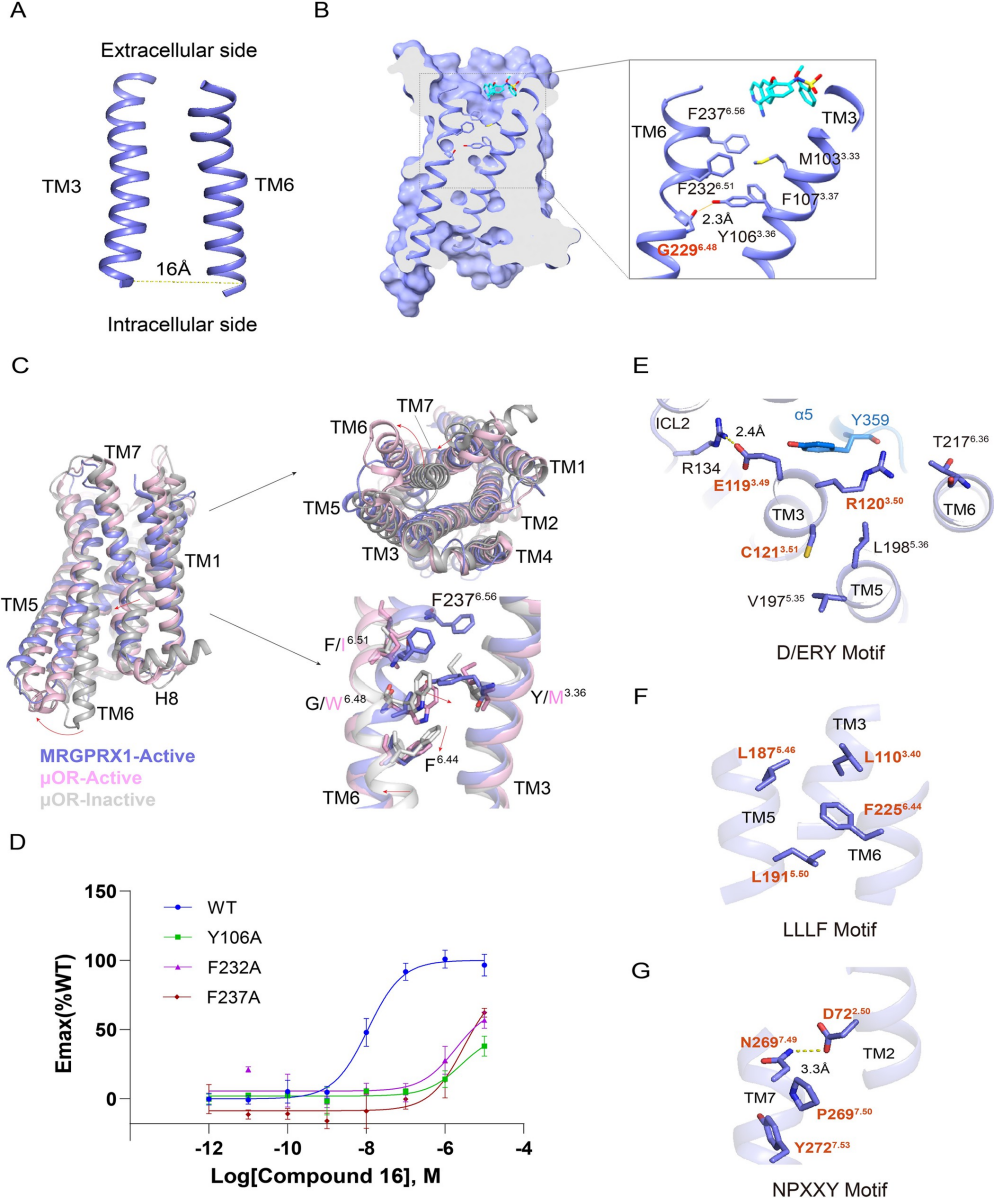
Activation mechanism of MRGPRX1 and conformational switches of MRGPRX1 in active state. (A) Structural representation of TMs arrangement between TM3 and TM6. The distance between the C-terminus of TM3 and TM6 is shown as a dashed line. (B) Structural representation of the interactions near the substituted toggle switch G229^6.48^. (C) Structural superposition of active MRGPRX1, active μOR (PDB 7U2L) [34] and inactive μOR (PDB 7UL4) [35] from the side, cytoplasmic, and magnified views. The movement directions of TM6, TM7, and residues in MRGPRX1 relative to inactive μOR are highlighted as red arrows. MRGPRX1, active μOR, and inactive μOR are colored in slate, light pink, and gray, respectively. (D) BRET validation of essential residues in the extracellular half of TM6. Data are presented as mean ± SEM. *n* = 3; Emax, maximum effect; WT, wild type. Magnified view of D/ERY motif (E), LLLF motif (F), and NPXXY motif (G). Polar interactions are shown as yellow dashed lines. The underlying data for Fig 3D can be found in S1 Data. BRET, bioluminescence resonance energy transfer; μOR, μ opioid receptor.

The compound 16-MRGPRX1-G_αq_ complex structure exhibited TM rearrangement in the cytoplasmic half. The cytoplasmic ends of TM3 and TM6 are about 16 angstroms apart, consistent with other class A G protein-engaged GPCRs in an active conformation [33] (Fig 3A). Notably, the conserved toggle switch W^6.48^ in other GPCRs is replaced by G229^6.48^ in MRGPRX1. This vital substitution results in an inward movement of the extracellular half of TM6, narrowing the gap between TM3 and TM6 and initiating the formation of a shallow orthosteric pocket (Fig 3B). Briefly, Y106^3.36^ in TM3 engages with G229^6.48^ in TM6 to form a twist. This twist is then stabilized by the hydrophobic interactions network among Y106^3.36^, F232^6.51^, and F237^6.56^, which prevents the ligands from entering the deeper location and exhibits a shallow pocket to accommodate ligands. Additionally, we conducted a structural comparison of MRGPRX1 complex to its functional closely related μ opioid receptor (μOR) in the active state (PDB 7U2L) [34] and inactive state (PDB 7UL4) [35] (Fig 3C). The structural comparison demonstrates that the MRGPRX1 complex shows a similar structure as the active μOR. Moreover, the structure superposition of G_αq_-coupled MRGPRX1 with G_αq_-coupled 5-HT_2A_R (PDB 6WHA) [36] and G_αq_-coupled B1R (PDB 7EIB) [30] by receptors also exhibits similar conformations (S8A and S8B Fig), suggesting a common activation mechanism among these receptors. Significantly, except for the extracellular half of TM6, G_αq_-coupled MRGPRX1 shows nearly identical conformations of TM3, TM6, and TM7 with these receptors in active state (Figs 3C and S8). MRGPRX1 possesses several unique residues in its extracellular half of TM6, which are essential for receptor activation. Furthermore, consistent with our speculation, alanine mutations of these residues dramatically affected MRGPRX1 activation induced by compound 16 (Fig 3D). Hence, the initiation of MRGPRX1 activation is likely triggered by touching F237^6.56^ at the bottom of the pocket upon agonist binding, pushing a series of residues in TM6 to move towards TM3 and resulting in the conformational change of G229^6.48^. G229^6.48^ shifts to get close to Y106^3.36^, triggers the rotation of conserved F^6.44^, and further facilitates the intracellular half of TM6 moving outward to accommodate the downstream G protein (Figs 3C and S8).

In addition to the unique twist structure in TM6, MRGPRX1 also shows significant differences in some classic motifs for class A GPCR activation. Firstly, the conserved D(E)^3.49^R^3.50^Y^3.51^ motif on TM3 of most class A GPCRs forms an ionic lock in an inactive conformation and is broken upon activation [33]. In MRGPRX1, Y^3.51^ is replaced by C121^3.51^, E119^3.49^ interacts with R134^ICL2^ via a strong salt bridge, and R120^3.50^ interacts with T217^6.36^ to limit the movement of TM6 and Y359 on G_αq_ to stabilize the complex via polar interactions (Fig 3E). Secondly, the conserved motif P^5.50^I^3.40^F^6.44^ [37] is substituted by an L187^5.46^L191^5.50^L110^3.40^F225^6.44^ motif, constraining the conformation of TM3/5/6 (Fig 3F). However, the conserved N^7.49^P^7.50^XXY^7.53^ motif, which does not interact directly with G proteins but is essential for receptor activation [33], is conserved in MRGPRX1 (Fig 3G). The above structure features are highly conserved among MRGPRXs, suggesting the common activation mechanism despite distinct agonist recognition features [14,15].

### The coupling of G_αq_ to MRGPRX1

The coupling of G_αq_ to MRGPRX1 is mainly maintained by interacting with residues on TM2, TM3, TM5, TM6, and ICL2 (S9A Fig). The interface between G_αq_ and TMs comprises a series of hydrophobic residues, including F61^2.39^, V124^3.54^, I202^5.61^, L211^6.30^, and L214^6.33^ on the receptor and L352, L356, and L361 on the α5-helix of G_αq_ (S9B Fig). However, alanine substitutions of these hydrophobic residues have little effect on G_αq_ coupling activity (S9C Fig and S1 Table). Only I202^5.61^A partially reduces G_αq_ activation (S9C Fig and S1 Table), suggesting that the TM bundles are less critical for G_αq_ activation in MRGPRX1. Moreover, in most class A GPCRs, ICL2 does not interact with G protein directly. However, extensive interactions of ICL2 with the αN-helix and α5-helix of G_αq_ are observed in the MRGPRX1 structure (S9D Fig). Alanine substitutions of I128^ICL2^ and H133^ICL2^ nearly impair G_αq_ coupling activity (S9E and S9F Fig, S1 Table), indicating that ICL2 plays a crucial role in G_αq_ coupling. Similar G protein coupling interfaces are also observed in the complex structures of MRGPRX2 and MRGPRX4 previously reported (S10A and S10B Fig).

## Discussion

In this study, we used the NanoBiT strategy to determine the structure of compound 16-bound MRGPRX1 in complex with G_αq_ via cryo-EM. We compared our compound 16-MRGPRX1-G_αq_ complex structure to the recently reported MRGPRX1-G_αq_ complex structure contributed by Liu and colleagues [16]. The overall structures are similar (S11A Fig), but the significant difference is the orientation of the phenylmethyl group in compound 16 (S11B and S11C Fig). Due to the better ligand density in our structure, compound 16 could be accommodated well with our proposed conformation. In contrast, the ligand leaves a part of the phenyl group out of the density map when we use Liu’s structure to fit the density. Our structure reveals the common feature of shallow, broad, and wide-open orthosteric pockets in all MRGPRX members. We speculated that this shallow and broad pocket might easily accommodate various small compounds with distinct scaffolds. The less selectivity helps the receptor expand the ligand spectrum of itch sensation and facilitates the body’s quick response to the diverse exogenous stimulus.

**Fig 4:**
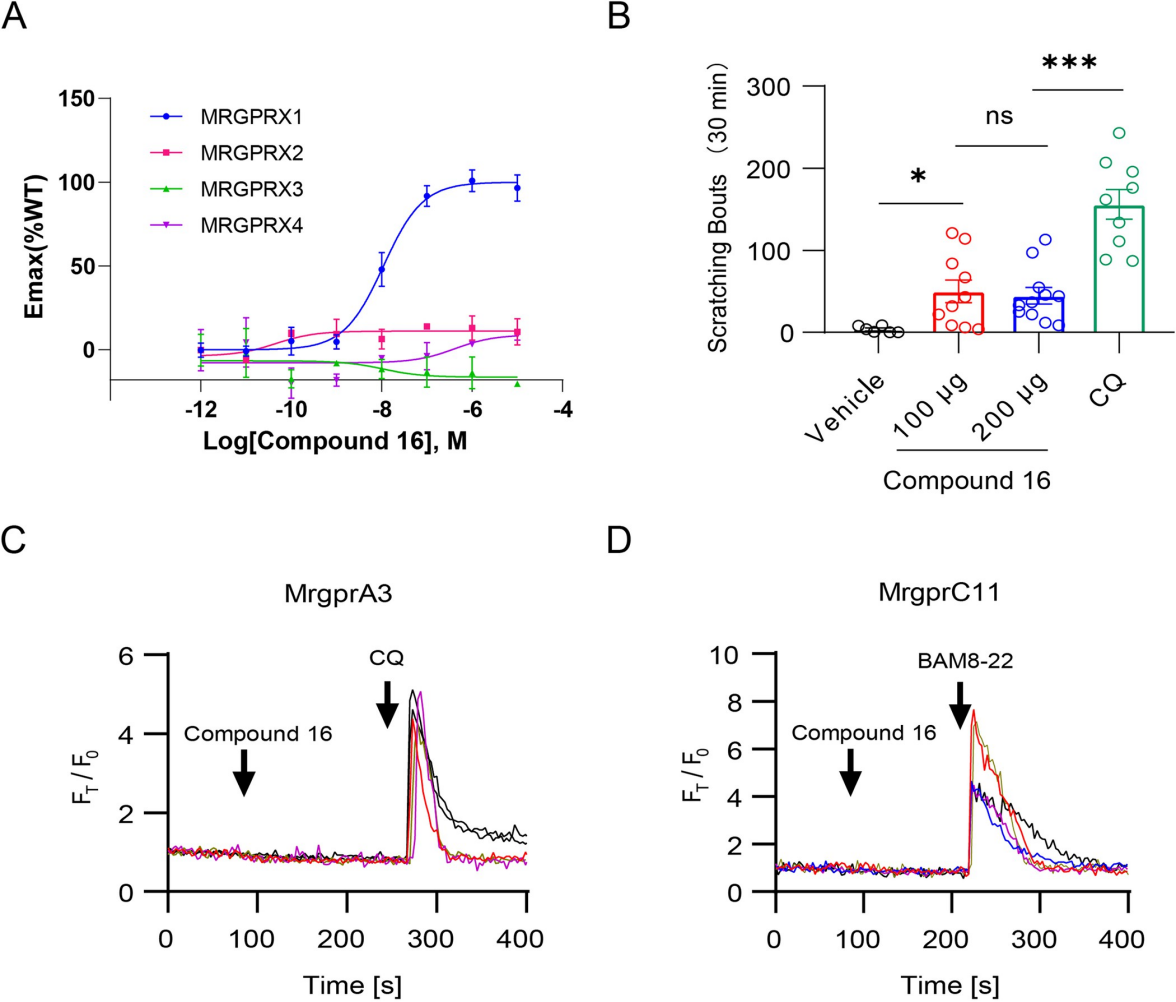
High selectivity of compound 16 to MRGPRX1 and its low itch risk. (A) Activation effect of compound 16 on MRGPRX1-X4. Data are presented as mean ± SEM. *n* = 3; Emax, maximum effect. (B) Itch behavior evaluation of compound 16. Scratching responses are induced by subcutaneous injection of vehicle (5% DMSO + 95% saline containing 20% SBE-β-CD, *n* = 6, 3.667 ± 1.647), compound 16 (100 μg, *n* = 10, 50.20 ± 13.93; 200 μg, *n* = 11, 44.82 ± 9.978) and CQ (200 μg, *n* = 9, 156.1 ± 18.24) in WT mice. Each dot represents an individual mouse. All data are presented as mean ± SEM. ns, not significant, *P* > 0.5; *, *P* < 0.05; ***, *P* < 0.001. (C) Representative calcium traces of MrgprA3 responding to compound 16 (10 μM). CQ (1 mM) was used as the positive control. (D) Representative calcium traces of MrgprC11 responding to compound 16 (10 μM). BAM8-22 (20 μM) was used as the positive control. The underlying data for Fig 4A–4D can be found in S1 Data. CQ, chloroquine; WT, wild type.

Notably, the binding site of compound 16 is closed to TM3 and TM4 of MRGPRX1, while the binding site of (R)-ZINC-3573 is closed to TM5 and TM6 of MRGPRX2 (Figs 2C and S12A). The conformation of ECL2 in MRGPRX2 may prevent the ligand access to the corresponding position in MRGPRX1 (S12B Fig). Similarly, the binding site of MS47134 is closed to TM2 and TM3 of MRGPRX4 (S12C Fig). The inward movement of TM3, TM4, and ECL2 in MRGPRX4 may prevent the ligand access to the corresponding position in MRGPRX1 (S12D Fig). Due to the distinct binding regions and the orthosteric pocket differences of these receptors, we evaluated the activation of compound 16 on MRGPRXs. As a result, MRGPRX1 is the only receptor that can be significantly activated by compound 16 with high potency (Figs 4A and S13A–S13I). All the above further confirms that the MRGPRXs differ in ligand recognition. Together with the highly conserved G protein interfaces among MRGPRXs, it can be concluded that MRGPRXs are activated by different ligands but use a general approach to recruit G proteins. These structural differences provide clues to design agonists with improved specificity and potency.

Moreover, MRGPRX1 reportedly involves in itch sensation [9] and pain inhibition [10]. Chloroquine (CQ), a drug widely used in malaria treatment, can cause itch in some people [38–40]. It is recently reported that MRGPRX1 mediates CQ-induced itch in humans [40]. Compound 16 was designed to inhibit chronic pain by targeting MRGPRX1. It is more abundant in the spinal cord than in the circulatory system, suggesting a lower risk of side effects caused by unexpected activation of MRGPRX1 [17]. Given its high potency, high selectivity, and restricted distribution, compound 16 is a viable candidate drug worthy of more attention and further study [17]. Accordingly, we tested the severity of itching that compound 16 might induce to evaluate whether the itch side effects would limit its application. Here, we used the scratching responses on the mouse model to evaluate the itch severity of compound 16 and CQ. Resultedly, compound 16 induces much less itch than a similar quantity of CQ (200 μg) (Fig 4B). To further investigate why compound 16 behaved differently than CQ, we tested the activation effect of compound 16 on MrgprA3 and MrgprC11, both the mouse orthologs of human MRGPRX1. Notably, MrgprA3 was found to be the main receptor mediating CQ-evoked responses in mice [40]. Our results showed that compound 16 could not activate MrgprA3 or MrgprC11 even at the concentration of 500 μM (Figs 4C and 4D and S13L and S13M). In other words, compound 16 failed to act as an agonist of mouse MrgprA3 and MrgprC11 but is a specific human MRGPRX1 agonist. Conversely, CQ shows higher potency to MrgprA3 (EC_50_: 27.55 μM) than MRGPRX1 (EC_50_: 297.68 μM) [40].

Determination of high-resolution CQ-bound MRGPRX1 and MrgprA3 structures may help us decipher the molecular basis of ligand recognition specificity. However, it is challenging to get a CQ-bound MRGPRX1 structure due to its low efficacy. Here, we performed molecular docking to analyze the recognition difference between MRGPRX1 and MrgprA3 for CQ (S14A and S14B Fig). The model of activated MrgprA3 was generated using MRGPRX1 as a template. Results show that CQ occupies a position away from TM6 in the binding pocket of MRGPRX1 compared with compound 16 in the MRGPRX1-compound 16 complex structure. It shows that the interactions between CQ and critical hydrophobic residues (F236^6.55^, F237^6.56^, and L240^6.59^) in TM6 are weaker than in compound 16. We speculate that CQ weakly activates MRGPRX1 due to its weak interactions with critical hydrophobic residues in TM6. Interestingly, the 7-chloroquinolin group of CQ in the pocket of MrgprA3 gets closer to TM6 when compared with that in MRGPRX1 structure. It is consistent with our speculation that stronger interactions with critical hydrophobic residues in TM6 will improve the ligand-binding affinity. Meanwhile, we also noticed several amino acids in the ligand-binding pocket that are unconserved between MRGPRX1 and MrgprA3. This may induce the different binding pose and affinity for compound 16 and CQ between MRGPRX1 and MrgprA3. Y99^3.29^ in MRGPRX1, critical for compound 16 binding, is replaced by a histidine in MrgprA3, which may affect the activation of MrgprA3 by compound 16.

Furthermore, studies on the downstream effectors of MRGPRX1, especially transient receptor potential vanilloid 1 (TRPV1) and transient receptor potential ankyrin 1 (TRPA1), show some conflicts. TRPV1 is usually regarded as an ion channel involved in pain sensation [41]. Wilson and colleagues found that TRPA1 is required for CQ-induced mice itch mediated by MrgprA3 [42]. The tick salivary peptide IPDef1 has been reported to evoke mice itch via MrgprC11 and result in the activation of the downstream ion channel TRPV1 rather than TRPA1 [43]. These data are consistent with the overlap between itch-sensing pathways and pain-sensing pathways. Thus, the differences in downstream signaling of MRGPRX1-mediated pain and itch sensations still need further investigation.

## Materials and methods

### Construct design

The full-length wild-type (WT) human MRGPRX1 (residues M1-S382) was cloned into pFastBac vector with an HA signal peptide, an N-terminal Flag tag, and a C-terminal 10×His tag. A BRIL [22] protein was fused at the N-terminus of MRGPRX1 to improve the expression of the receptor. LgBit [23] was fused at the C-terminus of MRGPRX1 to stabilize the whole MRGPRX1-Gαq complex. There was no more modification for the MRGPRX1 sequence. Prof. H. Eric Xu from Shanghai Institute of Materia Medica donated the engineered Gαq chimera plasmid. This engineered G_αq_ [29,30] was designed based on a mini-G_αs/q71_ [24,25] skeleton with the replacement of G_i1_ N-terminus and the insertion of G_αi1_ α-helical domain, thus providing possible binding sites for 2 antibody fragments scFv16 and Fab-G50 [26,27]. Additionally, 2 dominant-negative mutations (G203A and A326S) were introduced to decrease the affinity of nucleotide binding [28]. The WT G_β1γ2_ with HiBit [23] fused at the C-terminus of the β1 subunit was cloned into pFastBac-Dual vector. Ric8A is a molecular chaperone that has been reported to be essential for the biogenesis and signaling of G_αq_ subunits, and the involved functions include facilitating the proper folding of G_αq_ and promoting the formation of G_α_ guanine nucleotide-binding pocket [44–46]. The full-length Ric8A was cloned into pFastBac vector. For biochemical assay, the full-length WT MRGPRX1 and the MRGPRX1 point mutants were cloned into mammalian expression vector pCDNA 3.1 with an N-terminal Flag tag. All these constructs were generated with a standard PCR-based strategy and homologous recombination (CloneExpress One Step Cloning Kit, Vazyme).

### Expression and purification of scFv16

The scFv16 with a C-terminal 8×His tag was expressed in *Trichoplusia ni* Hi5 insect cells and purified precisely as previously described [26,32,47]. Briefly, the Hi5 insect cells (3.0 to 4.0 million cells per mL) were infected with the scFv16 virus produced by the Bac-to-Bac system (Invitrogen) for 96 h. Then, the medium was collected and pH balanced to pH 8.0 by adding Tris-base powder. Chelating agents were quenched by adding 1 mM nickel chloride and 5 mM calcium chloride. After incubation at room temperature (25°C) for 1 h by stirring constantly, the supernatant was isolated by centrifugation (4,750 rpm, 30 min, 4°C) and incubated with Ni-sepharose (GE Healthcare) for 1 h at room temperature with constant stirring. The resin was collected and then washed by washing buffer (20 mM HEPES (pH7.5), 100 mM NaCl, and 20 mM Imidazole). The protein was eluted by elution buffer (20 mM HEPES (pH 7.5), 100 mM NaCl, and 250 mM Imidazole) and then incubated with HRV-3C protease at 4°C for 2 h to remove the C-terminal 8×His tag. The cleaved protein was further loaded onto Superdex 200 Increase 10/300 GL column (GE Healthcare) with running buffer (20 mM HEPES (pH 7.5), 100 mM NaCl). ScFv16 peak fraction was collected, flash-frozen, and stored at −80°C until use.

### Expression and purification of Nb35

Nanobody-35 (Nb35) was expressed in the *Escherichia coli* BL21 (DE3) and purified as previously reported [30,31]. Nb35 was cultured in LB with 50 μg/mL ampicillin at 37°C, 220 rpm for about 3 h until OD_600_ reached 1.0. Then, IPTG was added to induce the protein expression at 25°C, 220 rpm with a final concentration 1 mM. Then, the *E*. *coli* bacteria were collected by centrifugation (4,000 rpm, 20 min, 4°C) after 16 h. The pellets were resuspended in lysis buffer (25 mM HEPES (pH 7.5), 150 mM NaCl, 1 mM PMSF) and disrupted by ultrasonication. The supernatant was isolated by centrifugation (14,000 rpm, 60 min, 4°C) and incubated with Ni-NTA resin (Qiagen) for 1 h. The resin was washed by washing buffer (25 mM HEPES (pH 7.5), 150 mM NaCl, and 25 mM Imidazole) and then eluted by elution buffer (25 mM HEPES (pH 7.5), 150 mM NaCl, and 250 mM Imidazole). The eluted protein was concentrated and loaded onto Superdex 200 Increase 10/300 GL column (GE Healthcare) with running buffer (25 mM HEPES (pH 7.5), 150 mM NaCl). The Nb35 monomeric fractions were pooled and stored at −80°C for further use.

### Expression and purification of MRGPRX1-G_αq_ complex

The complex was expressed in *Spodoptera frugiperda* (SF9) insect cells. Baculovirus preparation was accomplished based on the baculovirus system manual (Invitrogen). For expression, the SF9 cells were cultured in serum-free medium (UK1000, UNION-BIOTECH) to 2.0 million cells per mL and co-infected with BRIL-MRGPRX1-LgBit, engineered G_αq_, G_β1γ2_-HiBit, and Ric8A at a virus ratio 1:1:1:1 for 48 h. The complex cell pellets were resuspended in lysis buffer (25 mM HEPES (pH 7.5), 150 mM NaCl, 5% Glycerol, 10 mM MgCl_2_, 20 mM KCl, 5 mM CaCl_2_) supplemented with Protease Inhibitors (1 mM PMSF, 2 μg/mL Aprotinin, 5 μg/mL Leupeptin, and 1 μg/mL Pepstatin). The suspension was homogenized and incubated with 10 μM compound 16 (MCE), scFv16 (20 μg/mL), Nb35 (15 μg/mL), and apyrase (25 mU/mL, NEB) for 1 h at room temperature. Then, 0.5% (w/v) lauryl maltose neopentylglycol (LMNG) (Anatrace) and 0.05% (w/v) CHS (Anatrace) was added into the suspension to solubilize the membrane. After extracting for 2 h at 4°C, the supernatant was isolated by centrifugation (18,000 rpm, 40 min, 4°C). The flag resin was washed with 10 column volumes washing buffer 1 (25 mM HEPES (pH 7.5), 150 mM NaCl, 5% Glycerol, 5 mM MgCl_2_, 5 mM CaCl_2_, 10 μM compound 16, 0.1% (w/v) LMNG, and 0.01% (w/v) CHS) and then washed with 10 column volumes washing buffer 2 (25 mM HEPES (pH 7.5), 150 mM NaCl, 5% Glycerol, 5 mM MgCl_2_, 5mM CaCl_2_, 10 μM compound 16, 0.01% (w/v) LMNG, and 0.001% (w/v) CHS). The complex was eluted with elution buffer (25 mM HEPES (pH 7.5), 150 mM NaCl, 5% Glycerol, 5 mM MgCl_2_, 5mM CaCl_2_, 10 μM compound 16, 200 μg/mL Flag peptide (GenScript), 0.01% (w/v) LMNG, 0.005% GDN (Anatrace), and 0.001% (w/v) CHS). The final complex elution was concentrated to less than 2 mL using an Amicon Ultra Centrifugal Filter (MWCO 100 kDa) and incubated with 50 μM compound 16 for 1 h at 4°C. Then, the complex was subjected to Superdex 200 Increase 10/300 GL column (GE Healthcare) with running buffer (25 mM HEPES (pH 7.5), 150 mM NaCl, 2 mM MgCl_2_, 10 μM compound 16, 0.00075% (w/v) LMNG, 0.00025% (w/v) GDN, 0.000075% (w/v) CHS) to remove uncoupled components. The monomeric peak fractions were collected and concentrated to the final concentration 12 mg/mL for cryo-EM grid preparation.

### Cryo-EM sample preparation and data acquisition

The concentrated sample (3.5 μL) at a concentration of 12 mg/mL was applied to glow-discharged holey carbon-coated grids (Quantifoil 200 mesh, Au R1.2/1.3). The grids were blotted for 3.5 s and flash-frozen in liquid ethane using a Vitrobot (Mark IV, Thermo Fisher Scientific). Images were recorded on a 300 kV Titan Krios G3i electron microscope (Thermo Fisher Scientific) equipped with Gatan K3 Summit direct detector and a GIF Quantum energy filter (slit width 20 eV). Movie stacks were collected using SerialEM [48] in counting mode at a magnification of 105,000× with the corresponding pixel size of 0.85 Å. Movies stacked with 50 frames were exposed for 2 s. Two data sets of the same sample were collected, including 3,447 and 2,799 movies separately. Two data sets were recorded at a total dose of about 56.15 e/Å2 and 58.32 e/Å2, respectively. The defocus range was set from −1.0 μM to −2.0 μM.

### Data processing

Data processing was performed using cryoSPARC [49]. Movies frames were aligned using Patch motion. CTF estimation was performed using Patch CTF. Particles were first picked using a blob picker with partial micrographs. 2D templates were generated by 2D classification. Particle picking of all micrographs was performed by a template picker. A total of 6,687,388 particles from 6,246 micrographs were extracted using a box size of 288 pixels and cropped into 72 pixels. After 2 rounds of 2D classification and 1 round of ab initio reconstruction, 1,808,631 particles were selected and re-extracted using a box size of 288 pixels. Ab initio reconstruction using partial particles was performed, and 279,237 particles were removed, remaining 1,529,394 particles. After that, 1 round of nonuniform and local refinement was performed. One round of ab initio reconstruction was performed again, generating a new data set with 1,006,848 particles. Finally, 1 round of nonuniform and local refinement was performed, generating a 3.0 Å map.

### Model building and refinement

The initial complex model was built using the structure of MRGPRX2 (PDB code: 7S8N) and Nb35 (PDB code: 7F4H) as templates. Models are then fitted into the density map and manually adjusted and rebuilt in COOT [50]. The restraint files of compound 16 and CHS were generated by Phenix.elbow package [51]. The complete model was finally refined in Phenix using real-space refinement with secondary structure and geometry restraints [52] and COOT. Overfitting of the model was checked by refining the model using one of the 2 independent maps from gold-standard refinement and calculating FSC against both half maps [53]. The final model was validated using Molprobity [54] (S2 Table). Structural figures were prepared in PyMOL (https://pymol.org/2/), UCSF Chimera [55], and UCSF ChimeraX [56].

### Bioluminescence resonance energy transfer assay (BRET)

BRET assays [57] were performed as previously reported [47] to measure MRGPRX1-mediated G_q_ protein activation. Briefly, HEK293T cells (ATCCCRL-11268; mycoplasma free) were co-transfected at a 1:1:1:1 ratio of receptor: G_q_-Rluc8: G_β_: G_γ_-GFP2. After at least 18 h, transfected cells were harvested and reseeded in opaque white bottom 96-well assay plates (Beyotime) at a density of 30,000 to 50,000 cells per well (DMEM plus 10% FBS). The medium was removed after 24 h. Cells were incubated with 40 μL 7.5 μM coelenterazine 400a (Goldbio) in drug buffer (1× Hank’s balanced salt solution (HBSS), 20 mM HEPES (pH 7.4), 0.3% BSA) for 2 min, and then treated with 20 μL compounds prepared in drug buffer at serial concentration gradient for additional 5 min. Plates were read in an LB940 Mithras plate reader (Berthold Technologies) with 395-nm and 510-nm emission filters. BRET ratios were calculated as the ratio of GFP2 emission (510 nm) to Rluc8 emission (395 nm) and analyzed by GraphPad prism 8.0.

### Behavioral studies

WT C57BL/6J mice (8-week-old males) in acute itch behavioral tests were purchased from the Disease Control and Prevention Center of Hubei Province in China. Vehicle and MRGPRX1 agonists were subcutaneously injected into the nape of neck of mice after acclimatization. Scratching behavior in mice was observed for 30 min following injection. A bout of scratching was defined as a scratching movement with the hind paw directed at the area of the injection site. Then, the scratching bouts directing at the injected site were quantified.

### Ethics statement

All acute itch behavioral tests in mice were performed with subcutaneous injection of compounds and approved by the Animal Care and Ethical Committee of Wuhan University under the International Association for the Study of Itch guidelines (Approval number: WDSKY0201707-2). After itch experiments, all mice were humanely killed by carbon dioxide asphyxiation.

### Calcium imaging

HEK293T cells were loaded with 5 μM Fluo-4AM (Yeasen) for 30 to 45 min in the dark, supplemented with 0.01% Pluronic F-127 (wt/vol, Yeasen), in 1× HBSS containing 140 mM NaCl, 5 mM KCl, 10 mM HEPES, 2 mM CaCl_2_, 2 mM MgCl_2_, and 10 mM d-(+)-glucose (pH 7.4). After washing 3 times with HBSS, emission at 520 nm was detected from 488 nm excitation for Fluo-4AM. Data were analyzed from at least 3 repeated experiments at 80 to 200 cells.

### Molecular docking

Molecular docking was performed using AutoDock Vina [58,59]. Briefly, the structure of MRGPRX1-compound 16 and the model of MrgprA3 using homology modeling based on the structure of MRGPRX1-compound 16 were used to do CQ docking. Homology modeling of MrgprA3 was performed using Swiss-model [60,61]. Open Babel was used to prepare the coordinates file of CQ and receptor with polar-hydrogens. A grid box with 25 × 25 × 25 grid points was used for searching. The results were checked, and the 2 top-ranking binding poses were selected. Figures were prepared in PyMOL (https://pymol.org/2/).

## Supporting information

S1 Fig Construct for MRGPRX1, G_β_-HiBit, and G_αq_ chimera construct used in this study. (A) Schematic representation of MRGPRX1 construct. HA signal peptide (reddish brown), Flag tag (pink), HRV-3C protease cleavage sites (deepsalmon), fusion protein BRIL (greencyan), MRGPRX1 (slate), LgBit (wheat), His tag (violet), and linker (gray). (B) Schematic representation of G_β_-HiBit construct. His tag (violet), HRV-3C protease cleavage sites (deepsalmon), G_β_ (light blue), HiBit (orange), and linker (gray). (C) Potency evaluation of the compound 16 induced G_αq_ dissociation in MRGPRX1-WT and BRIL-MRGPRX1-LgBit overexpressing cells. Data are presented as mean ± SEM. *n* = 3; Emax, maximum effect; WT, wild type. (D) Schematic representation of G_αq_ chimera construct. The skeleton of G_αq_ chimera is based on mini-G_αs/q71_, which is shown in magenta. The N-terminus in green is replaced by G_αi1_-N terminus (for scFv16 binding). G_αi_-AHD (deep olive) is inserted subsequently for Fab-G50 binding. Two dominant-negative mutations for decreasing the affinity of nucleotide binding are shown in cyan. The underlying data for S1C Fig can be found in S1 Data.
(TIF)

S2 Fig Compound 16-MRGPRX1-G_αq_ complex purification. (A) SDS-PAGE of the Flag-purified compound 16-MRGPRX1-G_αq_ complex. (B) Final size exclusion chromatography elution profile of the complex. The underlying data for S2A Fig can be found in S1 Raw Image.
(TIF)

S3 Fig Structural determination of compound 16 bound MRGPRX1-G_αq_ complex. (A) Representative micrograph and 2D classes. (B) Flowchart for cryo-EM data processing. Details can be found in the Materials and methods. (C) FSC curves of the final refined cryo-EM map. (D) Angular distribution of the particles used for the final reconstructions. (E) Local resolution of the final map estimated by cryoSPARC. (F) FSC between map and model. FSC curve of the final refined model against the full map, colored in blue. FSC curve of the model refined against the first half map against the same map, colored in orange. FSC curve of the model refined against the first half map against the second half map, colored in gray. (G) The density maps of the transmembrane helix of MRGPRX1, α5 helix and αN helix are shown as mesh.
(TIF)

S4 Fig Ligand-binding pocket of G_αq_-coupled MRGPRX1, MRGPRX2, and MRGPRX4. (A) Top view of the (R)-ZINC-3573-binding pocket from the extracellular side (surface mode). Pocket is colored gray, and (R)-ZINC-3573 is shown as yellow sticks. (B) Top view of the MS47134-binding pocket from the extracellular side (surface mode). Pocket is colored gray, and MS47134 is shown as magenta sticks. (C) Structural comparison of MRGPRX1-G_αq_-compound 16 complex with MRGPRX2-G_αq_-(R)-ZINC-3573 complex (PDB code: 7S8N) and MRGPRX4-G_αq_-MS47134 complex (PDB code: 7S8P) in an extracellular view (cartoon mode). MRGPRX1, MRGPRX2, and MRGPRX4 are colored slate, pale green, and wheat, respectively. Compound 16, (R)-ZINC-3573, and MS47134 are colored cyan, yellow, and magenta, respectively. (D) Electrostatic surface representation of the MRGPRX1 extracellular pocket calculated using PyMOL with compound 16 shown as cyan sticks. Red, negative; blue, positive. (E) Electrostatic surface representation of the MRGPRX2 extracellular pocket calculated using PyMOL with (R)-ZINC-3573 shown as yellow sticks. Red, negative; blue, positive. (F) Electrostatic surface representation of the MRGPRX2 extracellular pocket calculated using PyMOL with MS47134 shown as magenta sticks. Red, negative; blue, positive.
(TIF)

S5 Fig Sequence alignment of Human MRGPRX1, MRGPRX2, MRGPRX3, and MRGPRX4. The sequence alignment was created using Clustalw [62] and ESPript 3.0 servers [63]. TMs for MRGPRX1 receptor are shown as violet columns. The ligand-binding pocket residues discussed in the main text are shown as yellow-green asterisk.
(TIF)

S6 Fig Calcium imaging validation of the compound 16-binding pocket. Representative calcium traces of MRGPRX1-WT (A), K96A (B), Y99A (C), E157A (D), W158A (E), D177A (F), F236A (G), F237A (H), L240A (I), and W241A (J) responding to compound 16 (500 nM).
(TIF)

S7 Fig Detailed structure comparison of ligand-binding pocket among MRGPRX1, MRGPRX2, and MRGPRX4. (A) A comparison of the ligand-binding pocket between MRGPRX1 and MRGPRX2 in a magnifying view. The key residues in MRGPRX1 are colored slate, and the corresponding residues in MRGPRX2 are colored pale green. Compound 16 and (R)-ZINC-3573 are colored cyan and yellow, respectively. (B) A comparison of the ligand-binding pocket between MRGPRX1 and MRGPRX4 in a magnifying view. The key residues in MRGPRX1 are colored slate, and the corresponding residues in MRGPRX2 are colored wheat. Compound 16 and MS47134 are colored cyan and magenta, respectively.
(TIF)

S8 Fig Activation mechanism comparison of MRGPRX1 with G_αq_ coupled receptors. An overall conformational comparison of active MRGPRX1, active B1R (PDB 7EIB [30]), active 5-HT_2A_R (PDB 6WHA [36]), and inactive 5-HT_2A_R (PDB 6WH4 [36]) from the side (A), cytoplasmic (B), and magnified views (C). The movement directions of TM6, TM7, and residues in MRGPRX1 relative to inactive 5-HT_2A_R are highlighted as red arrows. MRGPRX1, active B1R, active 5-HT_2A_R, and inactive 5-HT_2A_R are colored in slate, teal, salmon, and blue white, respectively.
(TIF)

S9 Fig The coupling of MRGPRX1 to G_αq_. (A) The interface between the MRGPRX1 and G_αq_ from the overall view. (B) The detailed view of hydrophobic interaction of TMs of MRGPRX1 and α5-helix of G_αq._ (C) BRET validation of residues in the TM-α5 helix interface. (D) The detailed view of interaction of ICL2 of MRGPRX1 and G_αq._ (E, F) BRET validation of residues in ICL2-α5 helix and ICL2-αN helix interface. Data are presented as mean ± SEM. *n* = 3; Emax, maximum effect; WT, wild type. The underlying data for S9C, S9E and S9F Fig can be found in S1 Data.
(TIF)

S10 Fig Comparison of the engagement of α5 helix in structure of MRGPRX1, MRGPRX2, and MRGPRX4. (A) A comparison of the engagement of α5 helix to receptor between the MRGPRX1 and MRGPRX2, viewing from the TM5, TM3, and ICL2 front angle. MRGPRX1, MRGPRX2, α5 helix in MRGPRX1, and α5 helix in MRGPRX2 are colored slate, pale green, marine, and orange, respectively. (B) A comparison of the engagement of α5 helix to receptor between the MRGPRX1 and MRGPRX4, viewing from the TM5, TM3, and ICL2 front angle. MRGPRX1, MRGPRX4, α5 helix in MRGPRX1, and α5 helix in MRGPRX4 are colored slate, wheat, marine, and magenta, respectively.
(TIF)

S11 Fig Structure comparison of the compound 16-MRGPRX1-G_αq_ to the recently reported compound 16-MRGPRX1-G_αq_ (PDB 8DWH)_._ (A) Overall structure comparison of the 2 structures. (B, C) Comparison of ligand density in 2 structures.
(TIF)

S12 Fig Structural models of human MRGPRX2 and MRGPRX4 in an extracellular view. (A) Top view of the (R)-ZINC-3573-binding pocket from the extracellular side (cartoon mode). MRGPRX2 is colored pale green, and (R)-ZINC-3573 is shown as yellow sticks. (B) Top view of the superimposed MRGPRX1-compound 16 and MRGPRX2 (cartoon mode). MRGPRX1 is colored slate, and compound 16 is shown as cyan sticks. MRGPRX2 is colored pale green. (C) Top view of the MS47134-binding pocket from the extracellular side (cartoon mode). MRGPRX4 is colored wheat, and MS47134 is shown as magenta sticks. (D) Top view of the superimposed MRGPRX1-compound 16 and MRGPRX4 (cartoon mode). MRGPRX1 is colored slate, and compound 16 is shown as cyan sticks. MRGPRX4 is colored wheat.
(TIF)

S13 Fig Activation effect of compound 16 on human MRGPRXs and mouse orthologs. (A–D) Representative calcium traces of MRGPRX1 (A), MRGPRX2 (B), MRGPRX3 (C), and MRGPRX4 (D) responding to compound 16 (500 nM). Details are described in Materials and methods. (E) Representative calcium traces of MRGPRX1 responding to BAM8-22 (a positive agonist of MRGPRX1, 20 μM). (F–H) Representative calcium traces of MRGPRX2 (F), X3 (G), and X4 (H) responding to compound 16 (50 μM). C48/80 (the agonist of MRGPRX2, 20 μg/mL) and DCA (deoxycholic acid, the agonist of MRGPRX4, 20 μM) were used as positive controls. (I, J) Representative dose-response curves for the MRGPRX2 (I) and MRGPRX4 (J) receptors in BRET assay, C48/80 and DCA were used as positive controls. (K) Dose-response curve for HEK293T cells transfected with MRGPRX1 responding to different concentrations of compound 16 (0.1 nM, 1 nM, 10 nM, 50 nM, 100 nM, 500 nM, 1 μM, and 10 μM). (L) Dose-response curve for HEK293T cells transfected with MrgprA3 responding to different concentrations of compound 16 (500 nM, 1 μM, 10 μM, 100 μM, and 500 μM). (M) Dose-response curve for HEK293T cells transfected with MrgprC11 responding to different concentrations of compound16 (500 nM, 1 μM, 10 μM, 100 μM, and 500 μM). The underlying data for S13I–S13M Fig can be found in S1 Data.
(TIF)

S14 Fig Comparisons of different ligand-binding pockets from the magnified view. (A) A comparison between MRGPRX1-compound 16 and docking MRGPRX1 structure with CQ. (B) A comparison between MRGPRX1-compound 16 and docking MrgprA3 structure with CQ. Key residues and ligands are shown as sticks. MRGPRX1, docking MRGPRX1, and MrgprA3 are colored slate, pale green, and gray, respectively. Compound 16 and CQ are colored cyan and hot pink, respectively.
(TIF)

S15 Fig Raw data of the BRET assay. Dose-response curves comparison of WT with ligand-binding pocket residues (A), G_αq_ interface residues (B), and MRGPRXs (C). Data are presented as mean ± SEM. *n* = 3; WT, wild type. The underlying data for S15A–S15C Fig can be found in S1 Data.
(TIF)

S1 Table Statistics of BRET assay for MRGPRX1 mutants. (PDF)

S2 Table Cryo-EM data collection, refinement and validation statistics. (PDF)

S1 Data Underlying data for Figs 2D, 2G, 2H, 3D, 4A–4D, S1C, S2B, S9C, S9E, S9F, S13I–S13M and S15A–S15C. (XLSX)

S1 Raw Image Uncropped Coomassie-stained SDS-PAGE gel used for S2A Fig. (PDF)

## Abbreviations

BRET: bioluminescence resonance energy transfer
CQ: chloroquine
cryo-EM: cryo-electron microscopy
DRG: dorsal root ganglia
HBSS: Hank’s balanced salt solution
LMNG: lauryl maltose neopentylglycol
MRGPR: Mas-related G-protein-coupled receptor
TG: trigeminal ganglia
TRPA1: transient receptor potential ankyrin 1
TRPV1: transient receptor potential vanilloid 1
WT: wild type
μOR: μ opioid receptor
