# Supplementary figures and images for: Mechanism of agonist-induced activation of the human itch receptor MRGPRX1
Source: PLoS Biol. 2023 Jun 22;21(6):e3001975. doi: 10.1371/journal.pbio.3001975 (PMC10286997; doi:10.1371/journal.pbio.3001975)

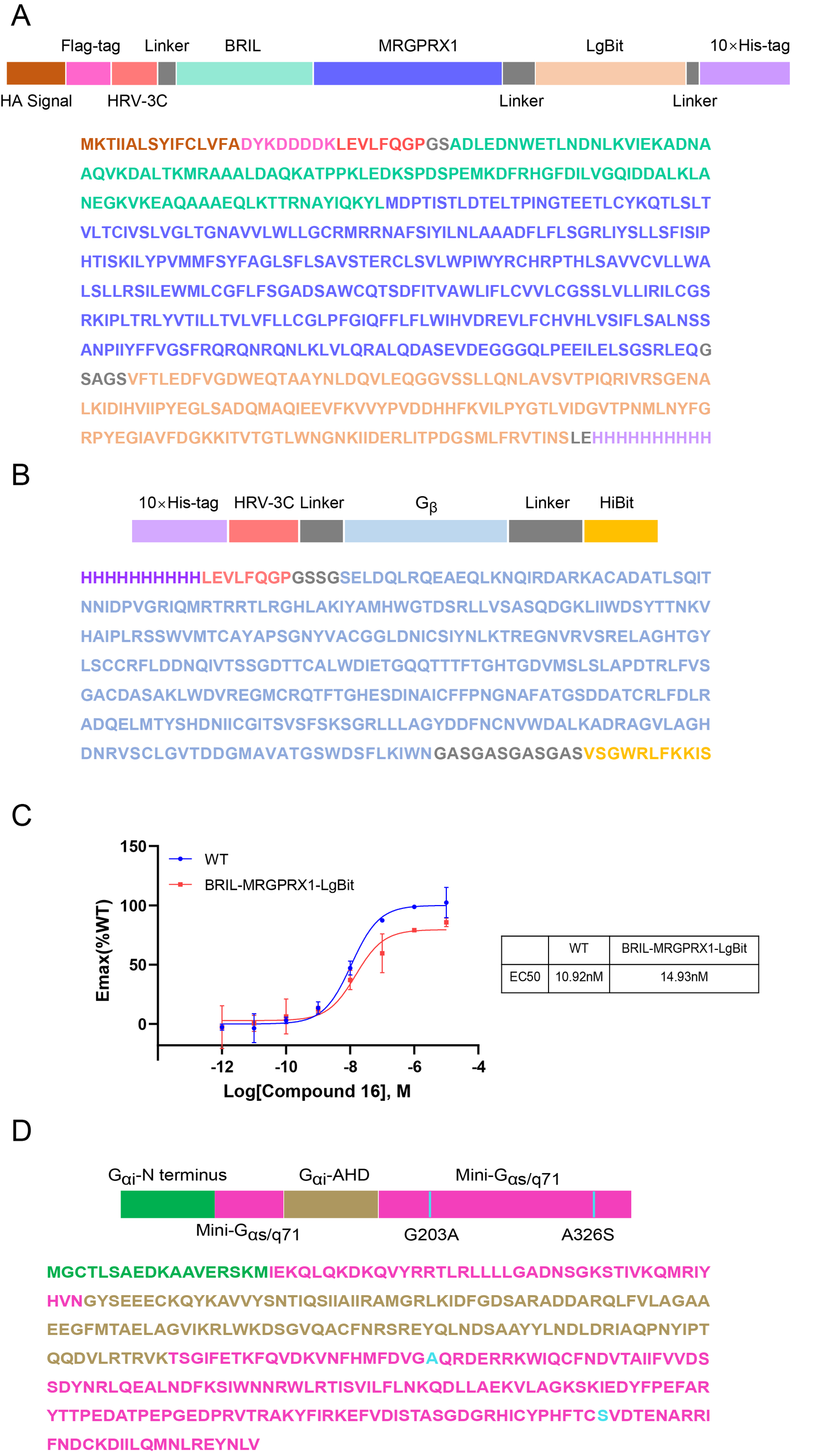

Supplement: S1 Fig — (A) Schematic representation of MRGPRX1 construct. HA signal peptide (reddish brown), Flag tag (pink), HRV-3C protease cleavage sites (deepsalmon), fusion protein BRIL (greencyan), MRGPRX1 (slate), LgBit (wheat), His tag (violet), and linker (gray). (B) Schematic representation of Gβ-HiBit construct. His tag (violet), HRV-3C protease cleavage sites (deepsalmon), Gβ (light blue), HiBit (orange), and linker (gray). (C) Potency evaluation of the compound 16 induced Gαq dissociation in MRGPRX1-WT and BRIL-MRGPRX1-LgBit overexpressing cells. Data are presented as mean ± SEM. n = 3; Emax, maximum effect; WT, wild type. (D) Schematic representation of Gαq chimera construct. The skeleton of Gαq chimera is based on mini-Gαs/q71, which is shown in magenta. The N-terminus in green is replaced by Gαi1-N terminus (for scFv16 binding). Gαi-AHD (deep olive) is inserted subsequently for Fab-G50 binding. Two dominant-negative mutations for decreasing the affinity of nucleotide binding are shown in cyan. The underlying data for S1C Fig can be found in S1 Data. (TIF) [file pbio.3001975.s001.tif]

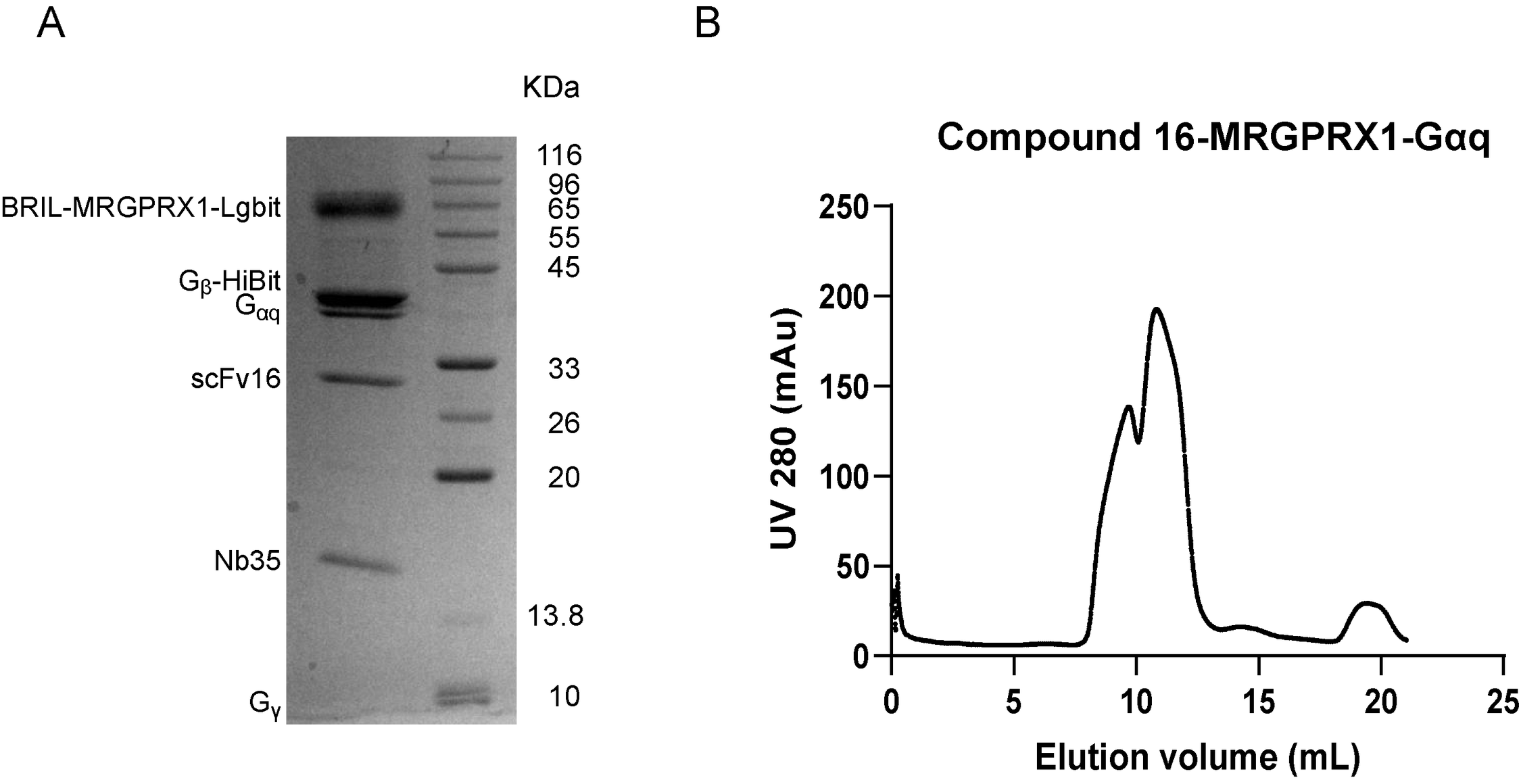

Supplement: S2 Fig — (A) SDS-PAGE of the Flag-purified compound 16-MRGPRX1-Gαq complex. (B) Final size exclusion chromatography elution profile of the complex. The underlying data for S2A Fig can be found in S1 Raw Image. (TIF) [file pbio.3001975.s002.tif]

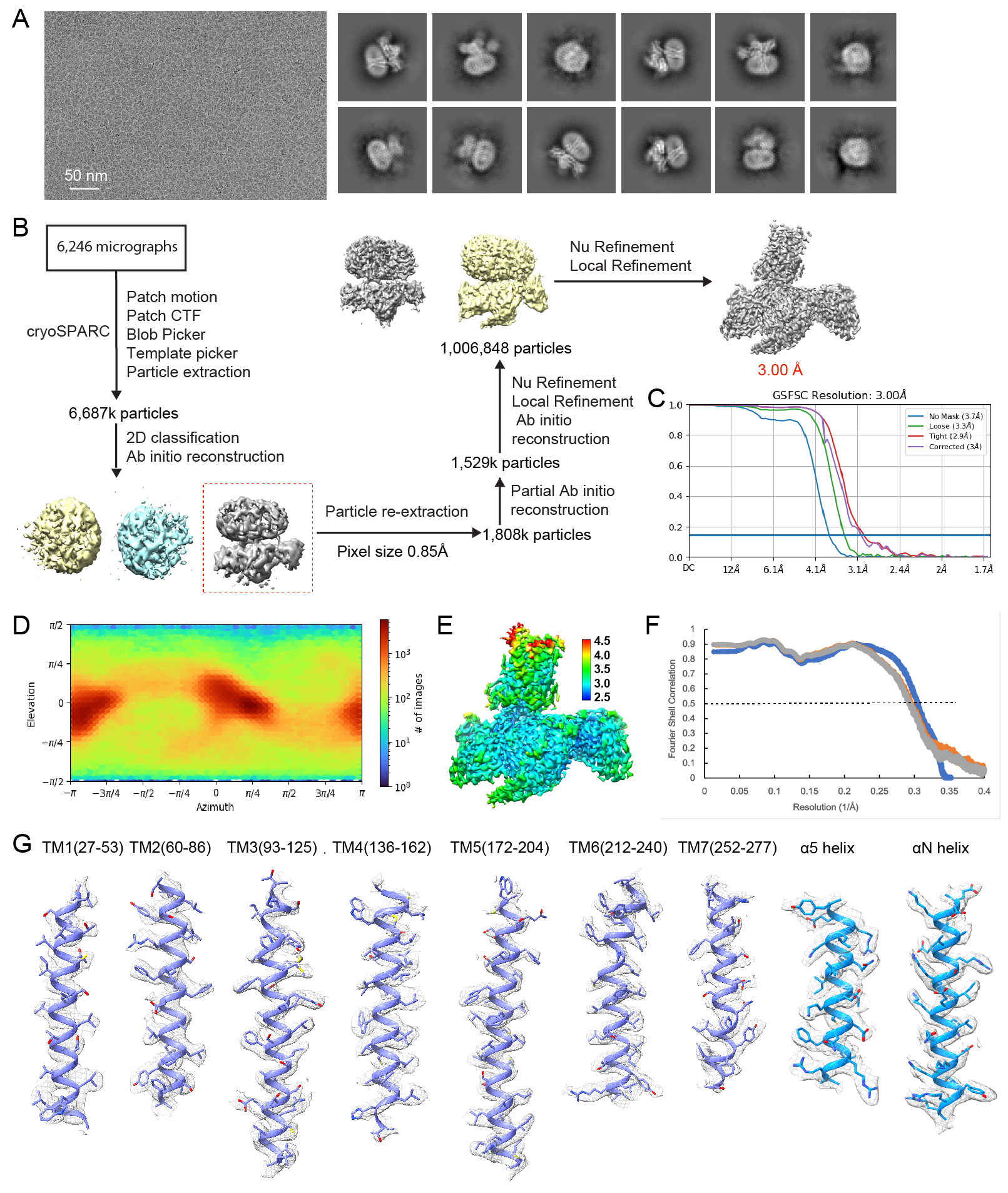

Supplement: S3 Fig — (A) Representative micrograph and 2D classes. (B) Flowchart for cryo-EM data processing. Details can be found in the Materials and methods. (C) FSC curves of the final refined cryo-EM map. (D) Angular distribution of the particles used for the final reconstructions. (E) Local resolution of the final map estimated by cryoSPARC. (F) FSC between map and model. FSC curve of the final refined model against the full map, colored in blue. FSC curve of the model refined against the first half map against the same map, colored in orange. FSC curve of the model refined against the first half map against the second half map, colored in gray. (G) The density maps of the transmembrane helix of MRGPRX1, α5 helix and αN helix are shown as mesh. (TIF) [file pbio.3001975.s003.tif]

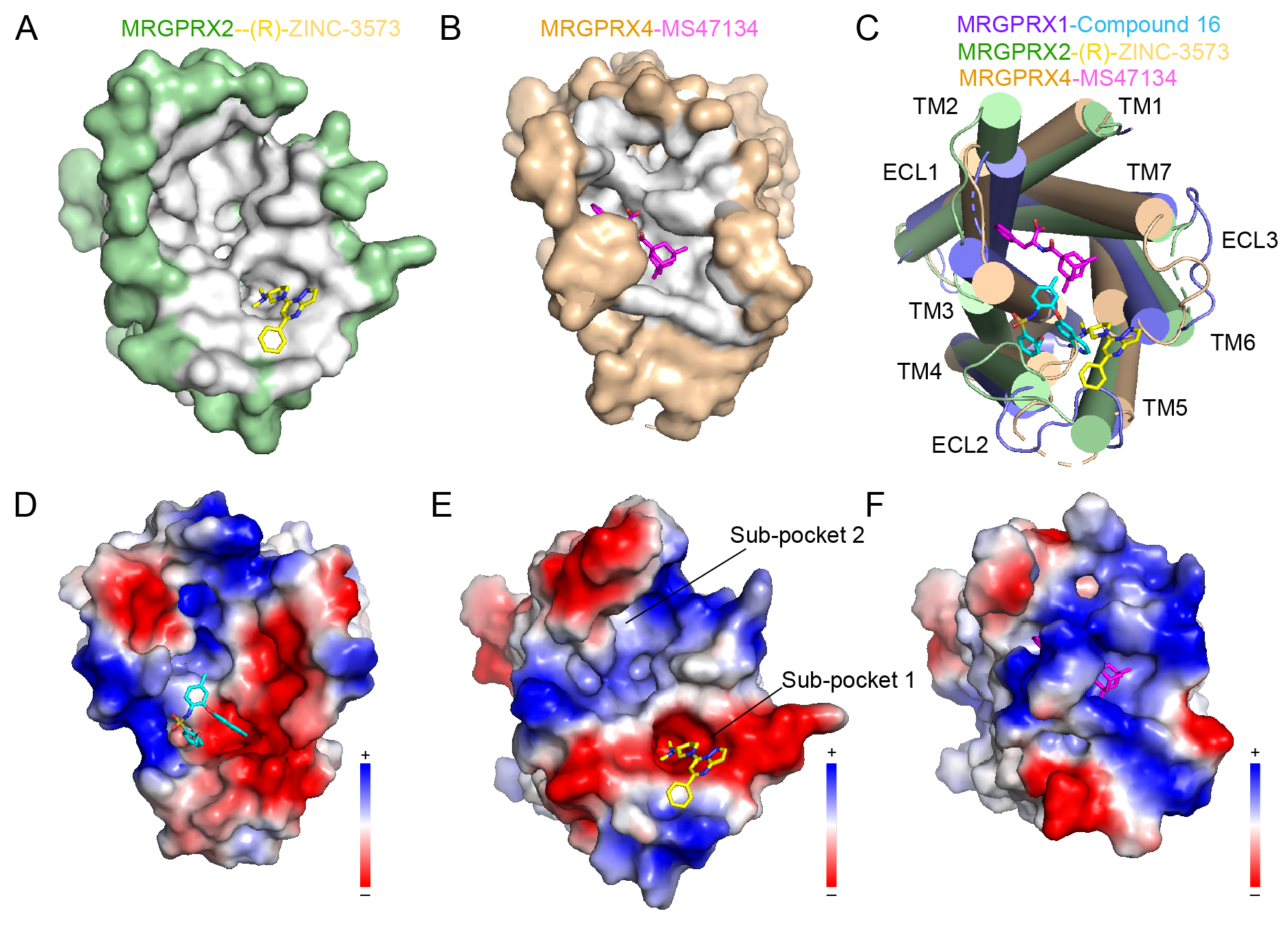

Supplement: S4 Fig — (A) Top view of the (R)-ZINC-3573-binding pocket from the extracellular side (surface mode). Pocket is colored gray, and (R)-ZINC-3573 is shown as yellow sticks. (B) Top view of the MS47134-binding pocket from the extracellular side (surface mode). Pocket is colored gray, and MS47134 is shown as magenta sticks. (C) Structural comparison of MRGPRX1-Gαq-compound 16 complex with MRGPRX2-Gαq-(R)-ZINC-3573 complex (PDB code: 7S8N) and MRGPRX4-Gαq-MS47134 complex (PDB code: 7S8P) in an extracellular view (cartoon mode). MRGPRX1, MRGPRX2, and MRGPRX4 are colored slate, pale green, and wheat, respectively. Compound 16, (R)-ZINC-3573, and MS47134 are colored cyan, yellow, and magenta, respectively. (D) Electrostatic surface representation of the MRGPRX1 extracellular pocket calculated using PyMOL with compound 16 shown as cyan sticks. Red, negative; blue, positive. (E) Electrostatic surface representation of the MRGPRX2 extracellular pocket calculated using PyMOL with (R)-ZINC-3573 shown as yellow sticks. Red, negative; blue, positive. (F) Electrostatic surface representation of the MRGPRX2 extracellular pocket calculated using PyMOL with MS47134 shown as magenta sticks. Red, negative; blue, positive. (TIF) [file pbio.3001975.s004.tif]

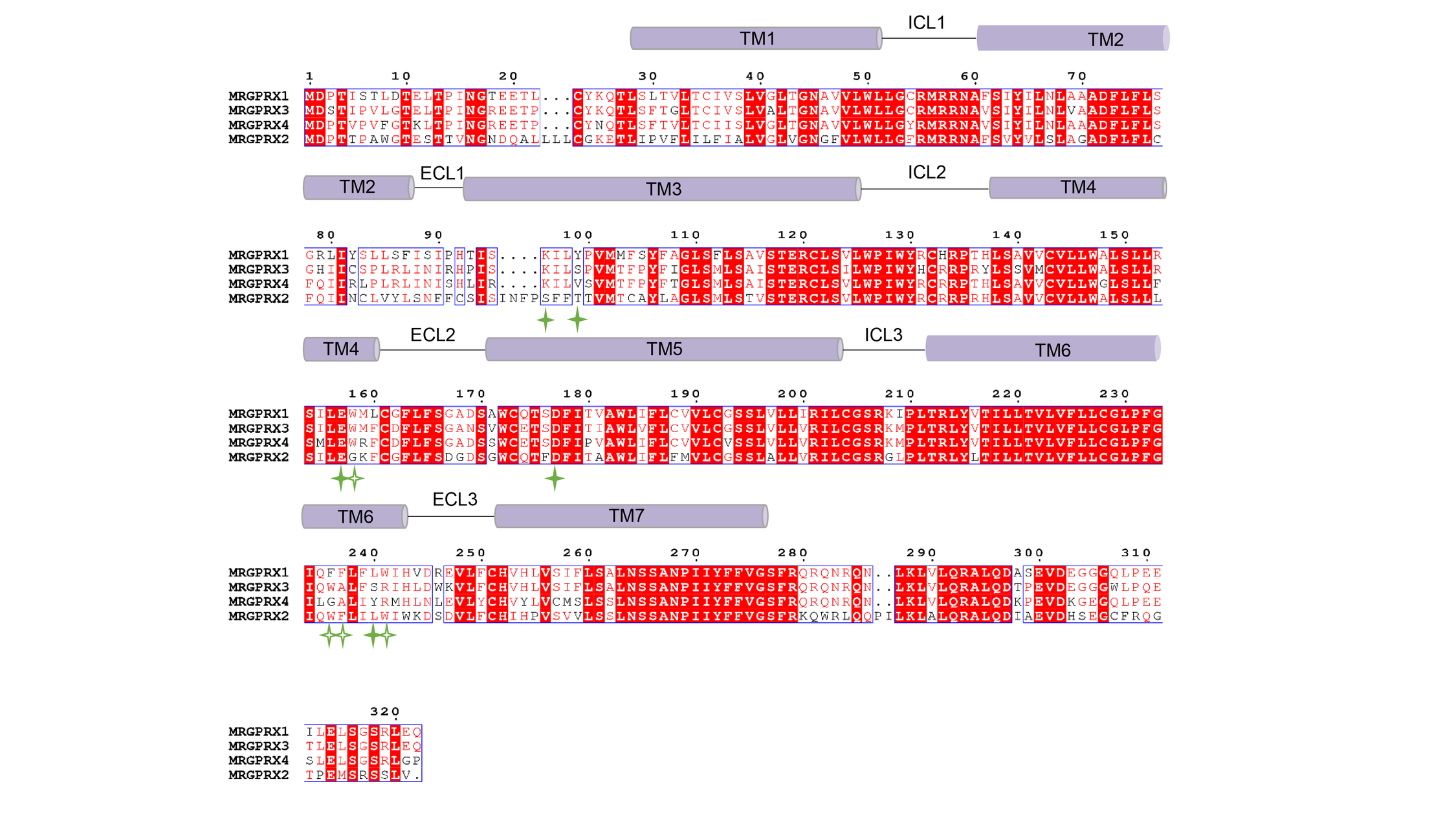

Supplement: S5 Fig — The sequence alignment was created using Clustalw [62] and ESPript 3.0 servers [63]. TMs for MRGPRX1 receptor are shown as violet columns. The ligand-binding pocket residues discussed in the main text are shown as yellow-green asterisk. (TIF) [file pbio.3001975.s005.tif]

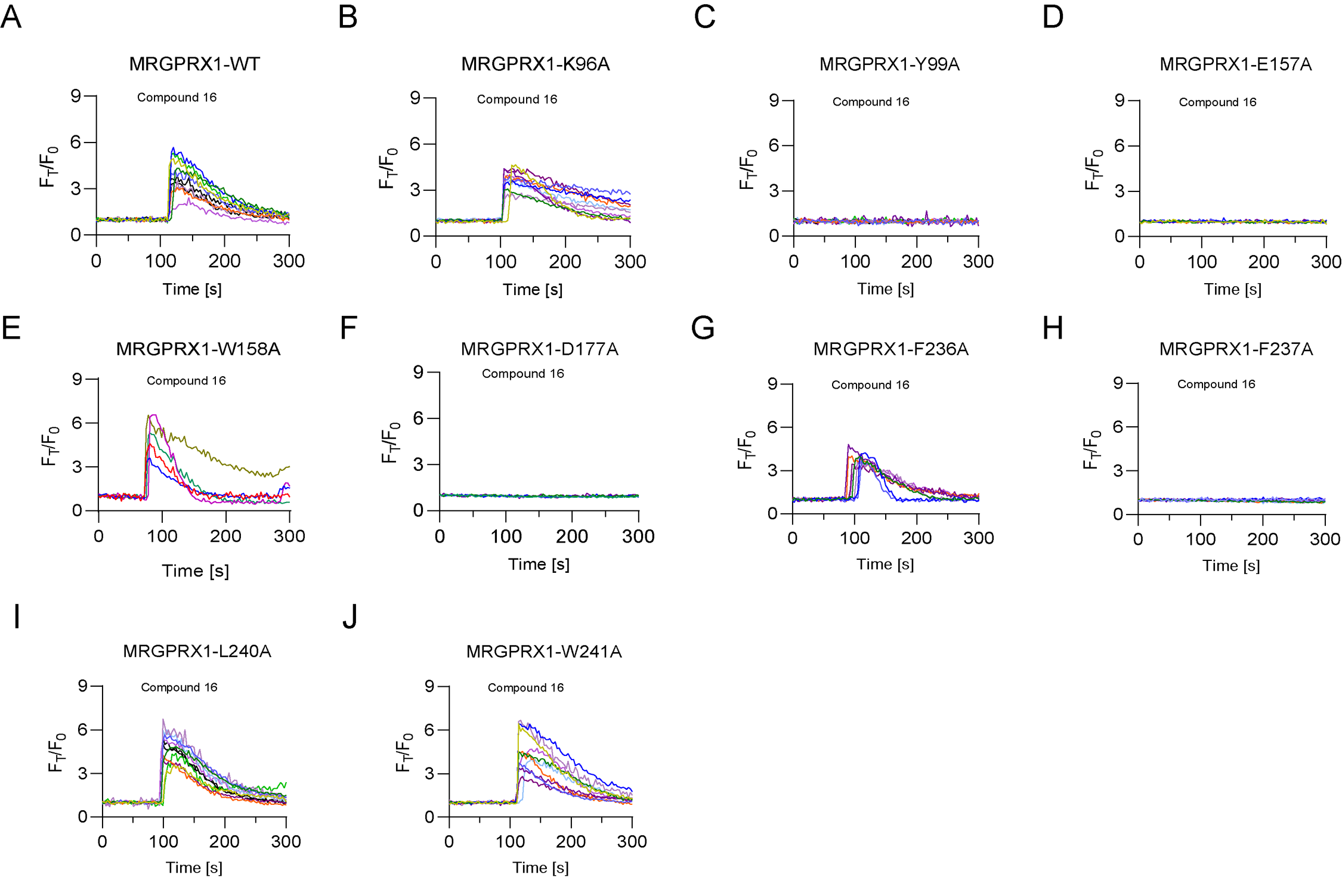

Supplement: S6 Fig — Representative calcium traces of MRGPRX1-WT (A), K96A (B), Y99A (C), E157A (D), W158A (E), D177A (F), F236A (G), F237A (H), L240A (I), and W241A (J) responding to compound 16 (500 nM). (TIF) [file pbio.3001975.s006.tif]

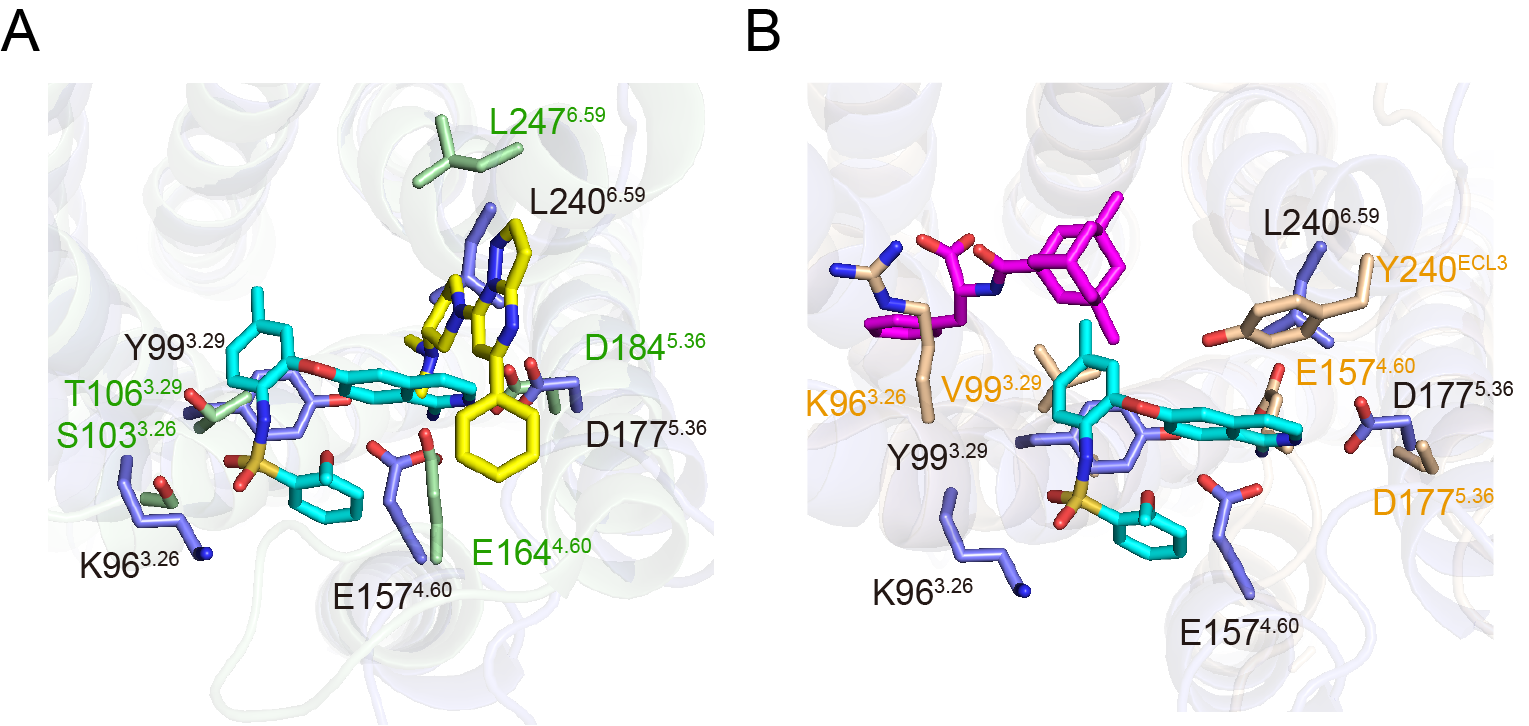

Supplement: S7 Fig — (A) A comparison of the ligand-binding pocket between MRGPRX1 and MRGPRX2 in a magnifying view. The key residues in MRGPRX1 are colored slate, and the corresponding residues in MRGPRX2 are colored pale green. Compound 16 and (R)-ZINC-3573 are colored cyan and yellow, respectively. (B) A comparison of the ligand-binding pocket between MRGPRX1 and MRGPRX4 in a magnifying view. The key residues in MRGPRX1 are colored slate, and the corresponding residues in MRGPRX2 are colored wheat. Compound 16 and MS47134 are colored cyan and magenta, respectively. (TIF) [file pbio.3001975.s007.tif]

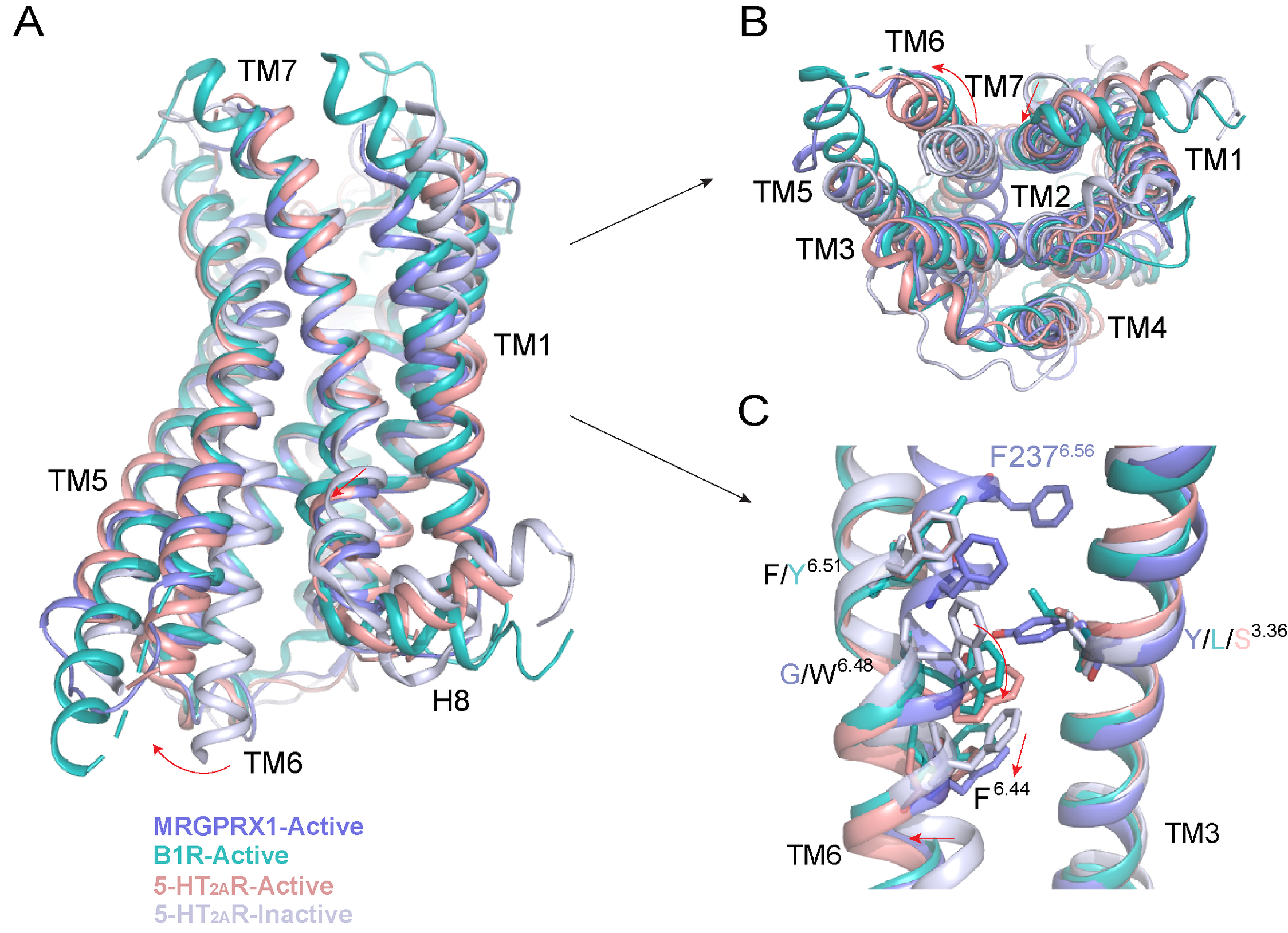

Supplement: S8 Fig — An overall conformational comparison of active MRGPRX1, active B1R (PDB 7EIB [30]), active 5-HT2AR (PDB 6WHA [36]), and inactive 5-HT2AR (PDB 6WH4 [36]) from the side (A), cytoplasmic (B), and magnified views (C). The movement directions of TM6, TM7, and residues in MRGPRX1 relative to inactive 5-HT2AR are highlighted as red arrows. MRGPRX1, active B1R, active 5-HT2AR, and inactive 5-HT2AR are colored in slate, teal, salmon, and blue white, respectively. (TIF) [file pbio.3001975.s008.tif]

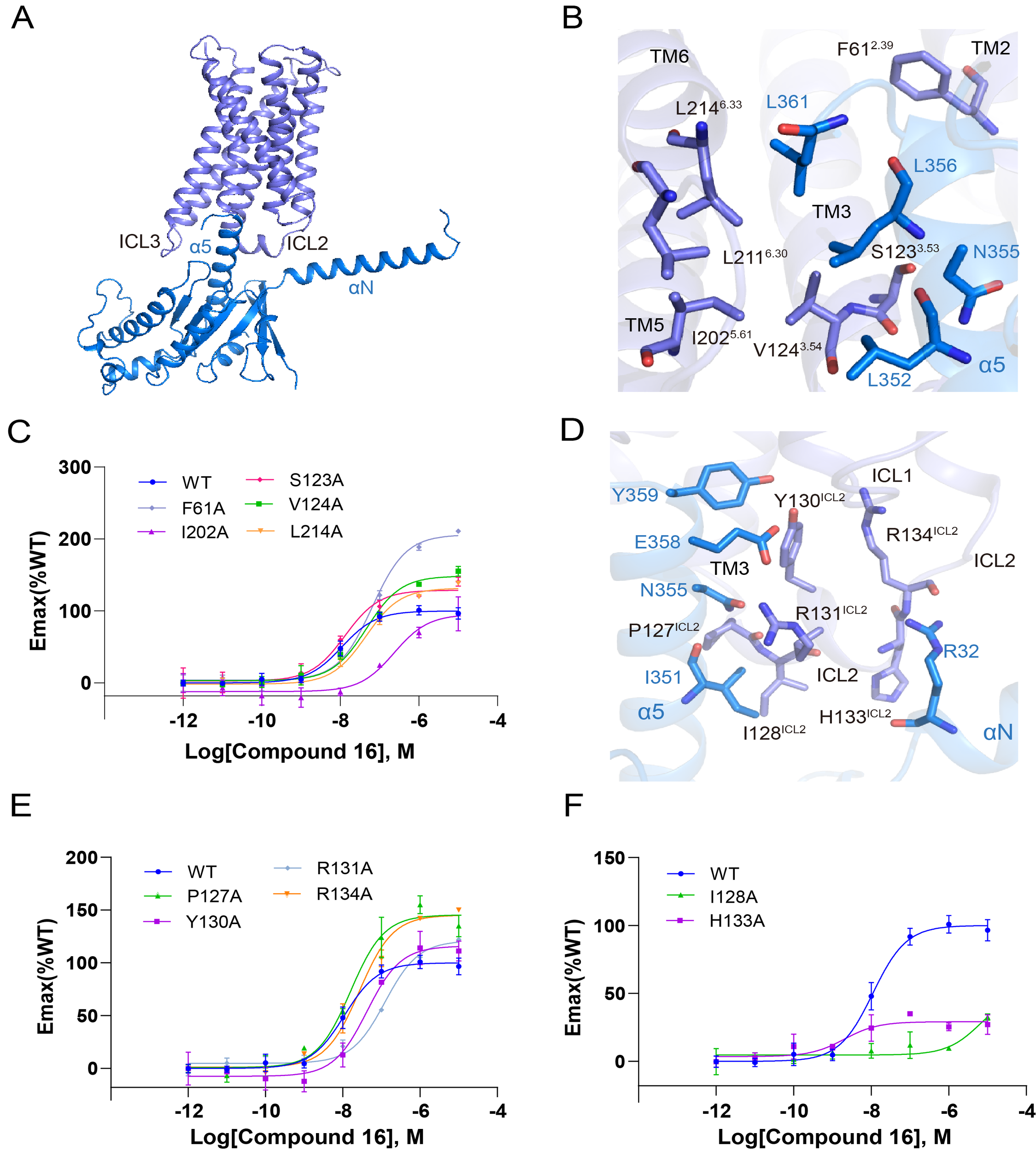

Supplement: S9 Fig — (A) The interface between the MRGPRX1 and Gαq from the overall view. (B) The detailed view of hydrophobic interaction of TMs of MRGPRX1 and α5-helix of Gαq. (C) BRET validation of residues in the TM-α5 helix interface. (D) The detailed view of interaction of ICL2 of MRGPRX1 and Gαq. (E, F) BRET validation of residues in ICL2-α5 helix and ICL2-αN helix interface. Data are presented as mean ± SEM. n = 3; Emax, maximum effect; WT, wild type. The underlying data for S9C, S9E and S9F Fig can be found in S1 Data. (TIF) [file pbio.3001975.s009.tif]

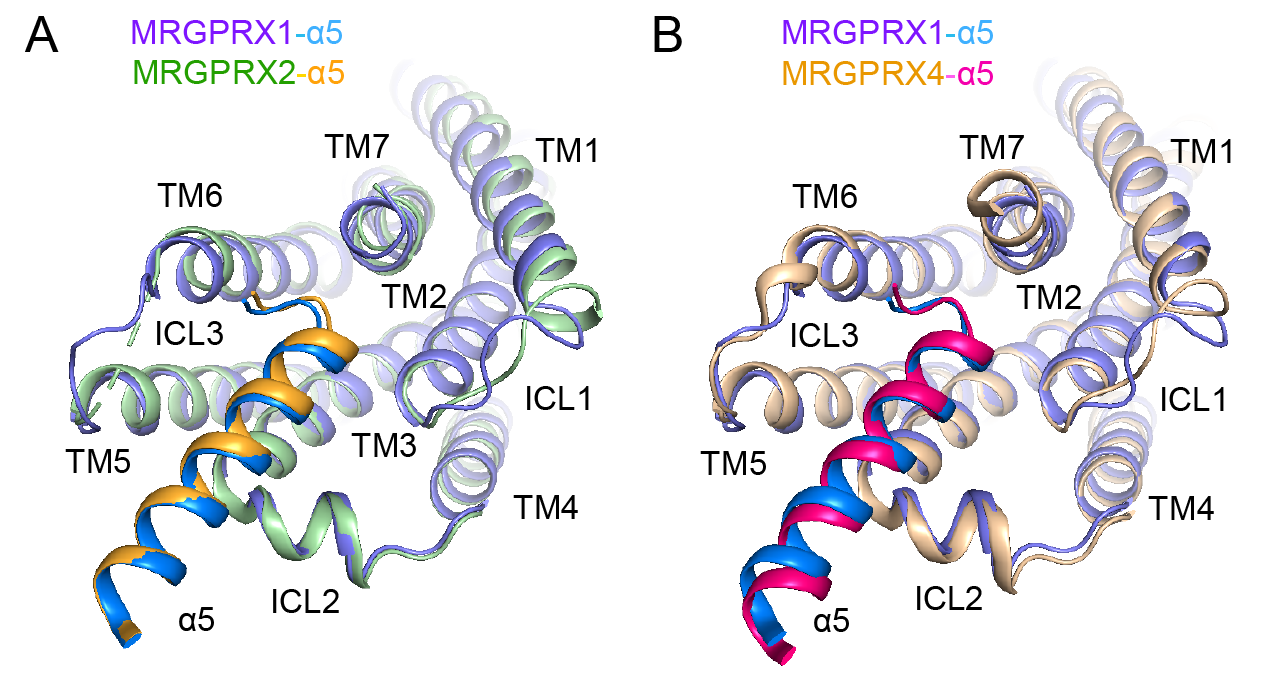

Supplement: S10 Fig — (A) A comparison of the engagement of α5 helix to receptor between the MRGPRX1 and MRGPRX2, viewing from the TM5, TM3, and ICL2 front angle. MRGPRX1, MRGPRX2, α5 helix in MRGPRX1, and α5 helix in MRGPRX2 are colored slate, pale green, marine, and orange, respectively. (B) A comparison of the engagement of α5 helix to receptor between the MRGPRX1 and MRGPRX4, viewing from the TM5, TM3, and ICL2 front angle. MRGPRX1, MRGPRX4, α5 helix in MRGPRX1, and α5 helix in MRGPRX4 are colored slate, wheat, marine, and magenta, respectively. (TIF) [file pbio.3001975.s010.tif]

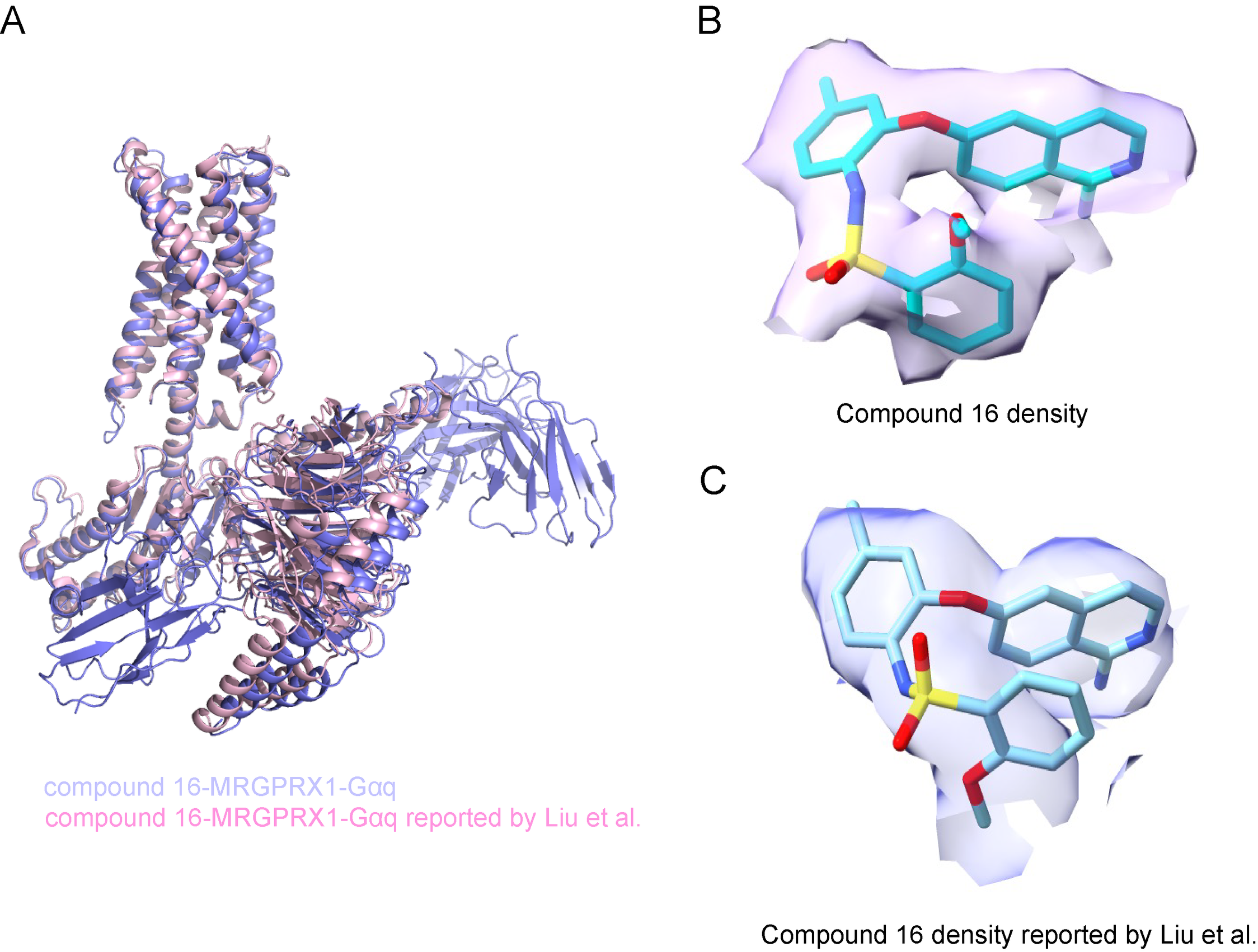

Supplement: S11 Fig — (A) Overall structure comparison of the 2 structures. (B, C) Comparison of ligand density in 2 structures. (TIF) [file pbio.3001975.s011.tif]

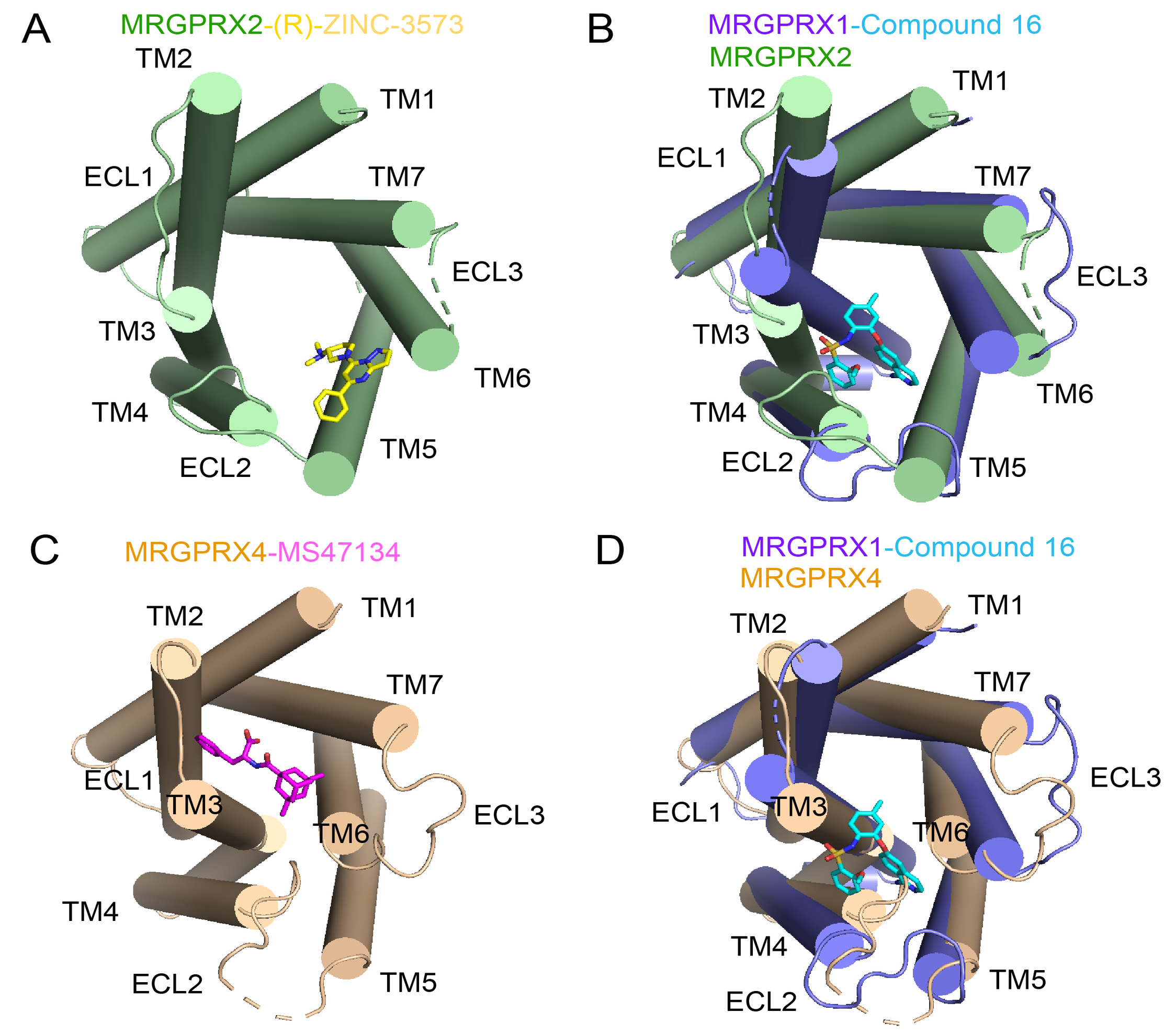

Supplement: S12 Fig — (A) Top view of the (R)-ZINC-3573-binding pocket from the extracellular side (cartoon mode). MRGPRX2 is colored pale green, and (R)-ZINC-3573 is shown as yellow sticks. (B) Top view of the superimposed MRGPRX1-compound 16 and MRGPRX2 (cartoon mode). MRGPRX1 is colored slate, and compound 16 is shown as cyan sticks. MRGPRX2 is colored pale green. (C) Top view of the MS47134-binding pocket from the extracellular side (cartoon mode). MRGPRX4 is colored wheat, and MS47134 is shown as magenta sticks. (D) Top view of the superimposed MRGPRX1-compound 16 and MRGPRX4 (cartoon mode). MRGPRX1 is colored slate, and compound 16 is shown as cyan sticks. MRGPRX4 is colored wheat. (TIF) [file pbio.3001975.s012.tif]

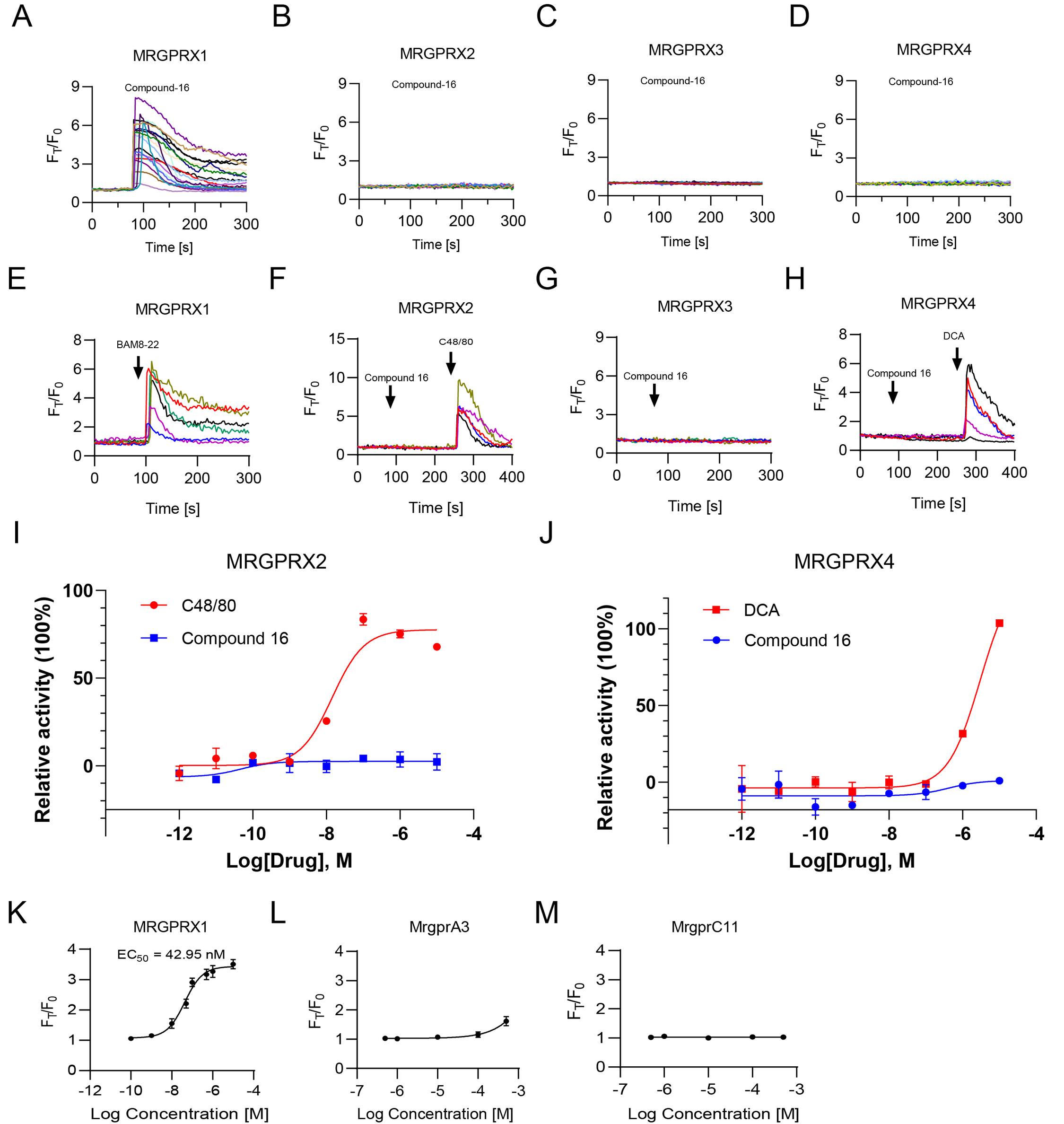

Supplement: S13 Fig — (A–D) Representative calcium traces of MRGPRX1 (A), MRGPRX2 (B), MRGPRX3 (C), and MRGPRX4 (D) responding to compound 16 (500 nM). Details are described in Materials and methods. (E) Representative calcium traces of MRGPRX1 responding to BAM8-22 (a positive agonist of MRGPRX1, 20 μM). (F–H) Representative calcium traces of MRGPRX2 (F), X3 (G), and X4 (H) responding to compound 16 (50 μM). C48/80 (the agonist of MRGPRX2, 20 μg/mL) and DCA (deoxycholic acid, the agonist of MRGPRX4, 20 μM) were used as positive controls. (I, J) Representative dose-response curves for the MRGPRX2 (I) and MRGPRX4 (J) receptors in BRET assay, C48/80 and DCA were used as positive controls. (K) Dose-response curve for HEK293T cells transfected with MRGPRX1 responding to different concentrations of compound 16 (0.1 nM, 1 nM, 10 nM, 50 nM, 100 nM, 500 nM, 1 μM, and 10 μM). (L) Dose-response curve for HEK293T cells transfected with MrgprA3 responding to different concentrations of compound 16 (500 nM, 1 μM, 10 μM, 100 μM, and 500 μM). (M) Dose-response curve for HEK293T cells transfected with MrgprC11 responding to different concentrations of compound16 (500 nM, 1 μM, 10 μM, 100 μM, and 500 μM). The underlying data for S13I–S13M Fig can be found in S1 Data. (TIF) [file pbio.3001975.s013.tif]

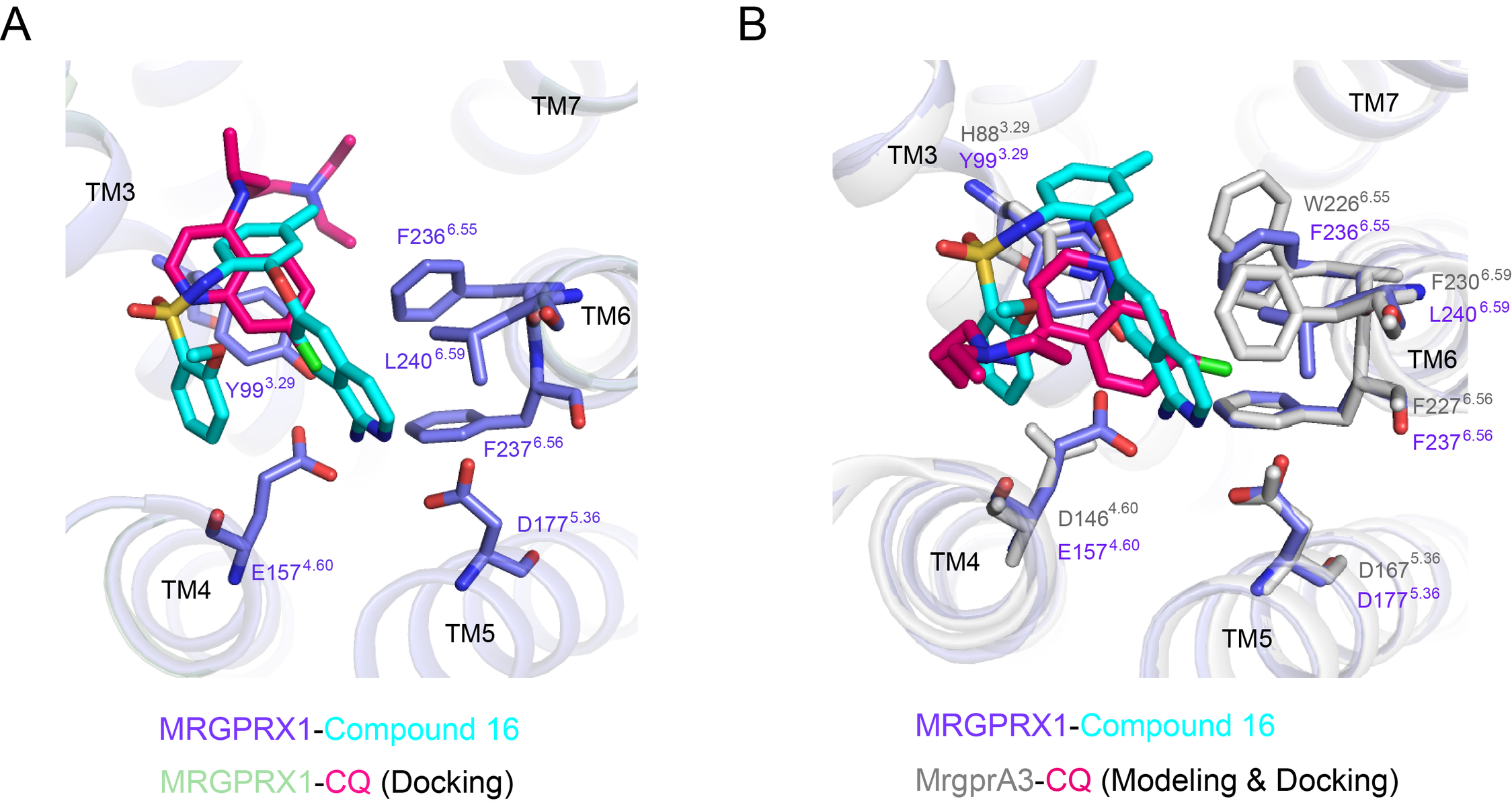

Supplement: S14 Fig — (A) A comparison between MRGPRX1-compound 16 and docking MRGPRX1 structure with CQ. (B) A comparison between MRGPRX1-compound 16 and docking MrgprA3 structure with CQ. Key residues and ligands are shown as sticks. MRGPRX1, docking MRGPRX1, and MrgprA3 are colored slate, pale green, and gray, respectively. Compound 16 and CQ are colored cyan and hot pink, respectively. (TIF) [file pbio.3001975.s014.tif]

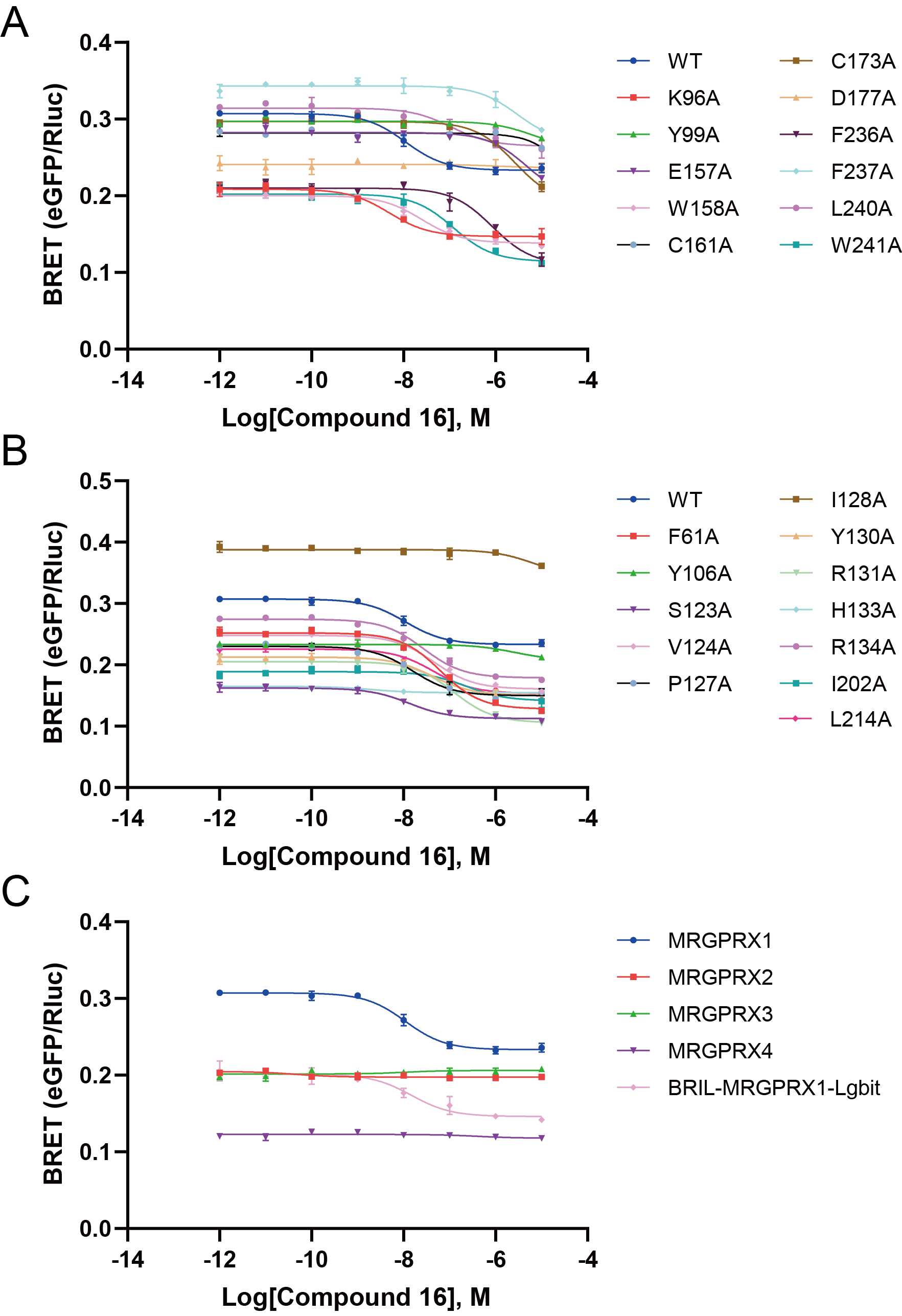

Supplement: S15 Fig — Dose-response curves comparison of WT with ligand-binding pocket residues (A), Gαq interface residues (B), and MRGPRXs (C). Data are presented as mean ± SEM. n = 3; WT, wild type. The underlying data for S15A–S15C Fig can be found in S1 Data. (TIF) [file pbio.3001975.s015.tif]
