# Supplementary material for: Mechanism of agonist-induced activation of the human itch receptor MRGPRX1
Source: PLoS Biol. 2023 Jun 22;21(6):e3001975. doi: 10.1371/journal.pbio.3001975 (PMC10286997; doi:10.1371/journal.pbio.3001975)
Supplement: S1 Table — (PDF) [file pbio.3001975.s016.pdf]

S1 Table. Statistics of BRET assay for MRGPRX1 mutants

MRGPRX1	Compound 16		
	EC_{50}, nM	pEC_{50} ± SEM	Fold
WT	10.92	7.96 ± 0.09	1.00
F61A	64.41	7.19 ± 0.06	5.90
K96A	4.87	8.31 ± 0.11	0.45
Y99A	6353	5.20 ± 0.61	581.78
Y106A	2599	5.59 ± 0.24	238.00
S123A	13.26	7.88 ± 0.12	1.21
V124A	46.65	7.33 ± 0.08	4.27
P127A	15.98	7.80 ± 0.10	1.46
I128A	6741	5.17 ± 0.70	617.31
Y130A	4.27	7.34 ± 0.12	0.39
R131A	124.6	6.91±0.07	11.41
H133A	ND	ND	/
R134A	26.98	7.57 ± 0.08	2.47
E157A	4937	5.31 ± 0.27	452.11
W158A	24.65	7.61 ± 0.11	2.26
C161A	ND	ND	/
C173A	2657	5.58 ± 0.10	243.32
D177A	ND	ND	/
I202A	234.1	6.63 ± 0.18	21.44
L214A	45.16	7.35 ± 0.09	4.14
F236A	849.6	6.07 ± 0.12	77.80
F237A	3018	5.52 ± 0.23	276.37
L240A	109.7	6.96 ± 0.24	10.05
W241A	121.9	6.9 ± 0.13	11.16
MRGPRX2	ND	ND	/
MRGPRX3	ND	ND	/
MRGPRX4	ND	ND	/
BRIL-MRGRX1-Lgbit	14.93	7.83 ± 0.19	1.37

The data are presented as mean ± s.e.m. n=3; Fold, the EC_{50} of mutant relative to the EC_{50} of WT; ND, no detected activity.
