# Supplementary material for: Mechanism of agonist-induced activation of the human itch receptor MRGPRX1
Source: PLoS Biol. 2023 Jun 22;21(6):e3001975. doi: 10.1371/journal.pbio.3001975 (PMC10286997; doi:10.1371/journal.pbio.3001975)
Supplement: S2 Table — (PDF) [file pbio.3001975.s017.pdf]

S2 Table. Cryo-EM data collection, refinement and validation statistics

	compound 16 bound<br>(EMDB-34833)<br>(PDB 8HJ5)
**Data collection and processing**	
Magnification	105,000
Voltage (kV)	300
Electron exposure (e–/Å^2)	56.15, 58.32
Defocus range (μm)	-1- -2
Pixel size (Å)	0.85
Symmetry imposed	C1
Initial particle images (no.)	6,687,338
Final particle images (no.)	1,006,848
Map resolution (Å)	3.0
FSC threshold	0.143
Map resolution range (Å)	2.5-4.5
**Refinement**	
Initial model used (PDB code)	7S8N, 7F4H
Model resolution (Å)	3.0/3.3
FSC threshold	0.143/0.5
Model composition	
Non-hydrogen atoms	9679
Protein residues	1232
Ligands	1
B factors (Å^2)	
Protein	94.81
Ligand	158.10
R.m.s. deviations	
Bond lengths (Å)	0.003
Bond angles (°)	0.660
Validation	
MolProbity score	1.81
Clashscore	8.11
Poor rotamers (%)	0.19
Ramachandran plot	
Favored (%)	94.65
Allowed (%)	5.35
Disallowed (%)	0
