# Supplementary material for: Mechanism of agonist-induced activation of the human itch receptor MRGPRX1
Source: PLoS Biol. 2023 Jun 22;21(6):e3001975. doi: 10.1371/journal.pbio.3001975 (PMC10286997; doi:10.1371/journal.pbio.3001975)
Supplement: S1 Raw Image — (PDF) [file pbio.3001975.s019.pdf]

Figure S2A

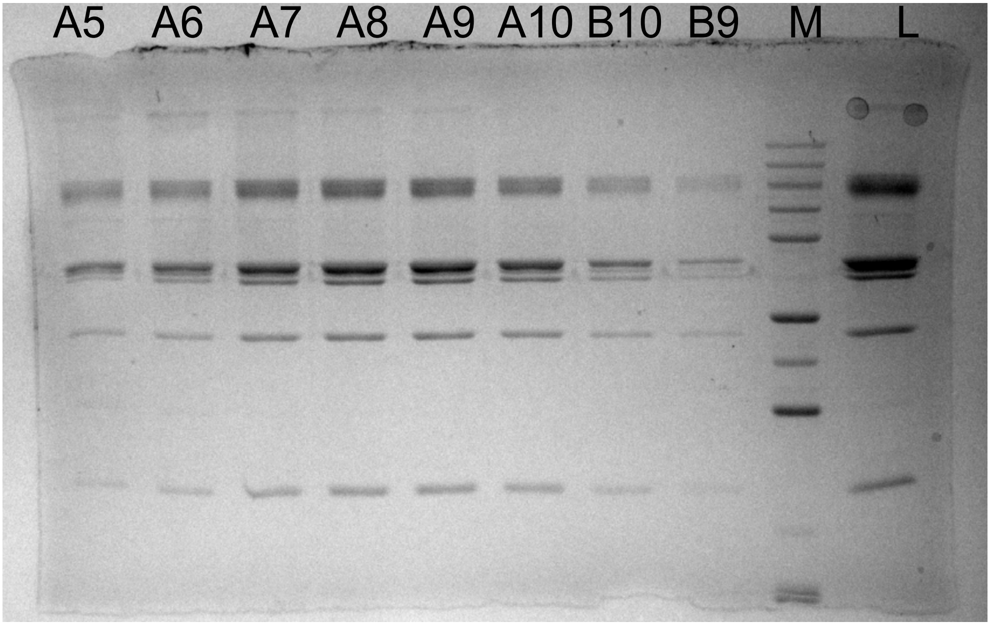

M = Marker (Protein ladder)
L = Sample loaded onto SEC
A5-B9 = Samples collected from SEC (Fraction numbers)
