# Supplementary material for: Mechanism of agonist-induced activation of the human itch receptor MRGPRX1
Source: PLoS Biol. 2023 Jun 22;21(6):e3001975. doi: 10.1371/journal.pbio.3001975 (PMC10286997; doi:10.1371/journal.pbio.3001975)
Supplement: Attachment — Submitted filename: R_20230428_Response to reviewers.docx [file pbio.3001975.s020.docx]

**Point-to-point response to Reviewer #1:**

***Reviewer #1:***

*This manuscript from Gan etc. reports a cryo-EM structure of the MRGPRX1-Gq complex with compound 16. The structure revealed a novel binding pose of compound 16 and a unique agonist recognition pattern compared to other MRGPRXs. Interestingly, the authors showed that compound 16 induced a much lowered itchy response compared to another MRGPRX1 agonist in an animal assay. The overall structural determination work is fine. The cryo-EM density map seems to allow unambiguous modeling. The results provide novel insight into MRGPR pharmacology.*

*1. One major issue is the lack of mutagenesis studies to support their structural findings. Considering the novel binding pose of compound 16, adequate mutagenesis studies are critical.*

**Response:** We thank the reviewer for this constructive suggestion. We confirmed the functions of key residues involved in the ligand-binding pocket by BERT assay and calcium imaging assay. A detailed description is added in the result part (Page 6-7). Figure 2 and Supplementary Figure 6 are modified. The summary table of BRET assay is also updated (Supplementary Table 1).

*2. Another major issue is the very limited discussion of their functional assay. Why compound 16 behaved differently compared to CQ? Do they induce different signaling pathways downstream of MRGPRX1?*

**Response:** We thank the reviewer for the insightful question. To further investigate why compound 16 behaved differently compared to CQ, we test the activation effects of compound 16 on MrgprA3 and MrgprC11. MrgprA3 and MrgprC11 are both the mouse orthologs of human MRGPRX1. Notably, MrgprA3 is the main receptor mediating CQ-evoked responses in mice[1]. Our result shows that neither MrgprA3 nor MrgprC11 can be activated by compound 16 even at the concentration of 500 μM, which explains why the conventional mouse model of itch can respond to CQ well but not respond to compound 16. In other words, the compound 16 is a specific human MRGPRX1 agonist with high selectivity among species. Its physiological functions could be investigated in a transgenic mouse line in which the genome fragment of mouse *Mrgprs* was replaced with human MrgprX1[2].

Additionally, CQ shows higher potency to MrgprA3 (EC_50_: 27.55 μM) than MRGPRX1 (EC_50_: 297.68 μM)[1]. We further performed molecular docking to analyze the recognition difference between MRGPRX1 and MrgprA3 for CQ. The model of activated MrgprA3 was generated using MRGPRX1 as a template. Results show that CQ occupies a position away from TM6 in the binding pocket of MRGPRX1 compared with compound 16 in the MRGPRX1-compound 16 complex structure. It shows that the interactions between CQ and critical hydrophobic residues (F236^6.55^, F237^6.56^, and L240^6.59^) in TM6 are weaker than compound 16. We speculate that CQ weakly activates MRGPRX1 due to its weak interactions with critical hydrophobic residues in TM6. Interestingly, the 7-chloroquinolin group of CQ in the pocket of MrgprA3 gets closer to TM6 when compared with that in MRGPRX1 structure. It is consistent with our speculation that stronger interactions with critical hydrophobic residues in TM6 will improve the ligand binding affinity. Meanwhile, we also noticed several amino acids in the ligand binding pocket that are not conserved between MRGPRX1 and MrgprA3, which may induce the different binding pose and affinity for compound 16 and CQ between MRGPRX1 and MrgprA3. Y99^3.29^ in MRGPRX1, critical for compound 16 binding, is replaced by a histidine in MrgprA3, which may affect the activation of MrgprA3 by compound 16.

All these supplementary results are updated in Figure 4, Supplementary Figure 13 and 14. The related description is added in the discussion part (Page 11, Line 12-29; Page 12, Line 1-8). We believe our results can answer the question why compound 16 behaves differently compared to CQ.

*Other minor issues:*

*1.* *It would be informative to discuss the potential mechanism by which compound 16 activates MRGPRX1. The authors only discussed the changes of some conserved microdomains in MRGPRX1.*

**Response:** We thank the reviewer for the crucial suggestion. Due to the lack of inactive MRGPRX1 structure, we compared our active MRGPRX1 structure with several well-studied GPCR structure, including the functional closely related μ opioid receptor (μOR) and G_αq_-coupled receptors (B1R & 5-HT_2A_R). The structure comparisons are shown in the updated Figure 3 and Supplementary Figure 8. We also propose a potential activation mechanism based on the results. The detailed mechanism description is added in the text (Page 8, Line 5-28).

*2. Figure 5A: What are the different traces? Also, does this figure just tell us compound 16 is a MRGPRX1 agonist? Any new information from this figure?*

**Response:** We are sorry for the ambiguity. Figure 5A just suggests that compound 16 is a MRGPRX1 agonist. Combined with the original Supplementary Figure 10, we try to illustrate that compound 16 is a specific MRGPRX1 agonist with high selectivity over MRGPRX2/3/4. To avoid misunderstanding, we double confirmed the activation effect of compound 16 on MRGPRXs by BRET assay and exchange the Figure 5A with the new BRET result including four curves in one single figure. The original Figure 5A is transferred to Supplementary Figure 13 and generate a new figure with original Supplementary Figure 10 for clear comparison.

Besides, to help understand the meaning of different traces, the principle of calcium imaging technology used in our study will be described first.

Ca^2+^ is the second messenger regulating the cell signaling and impacts nearly every aspect of cellular life. Cells affect signal transduction by regulating Ca^2+^ concentration. Underlying the speed and effectiveness of Ca^2+^ is the 20,000-fold gradient maintained by cells between their intracellular (~100 nM free) and extracellular (mM) concentrations. The cytoplasmic Ca^2+^ level is low in resting cells and is maintained at ~100 nM [3]. However, when the cells receive some stimuli leading to the activation of calcium channels and some GPCRs, the concentration of calcium in cytoplasm will increase rapidly. Hundreds of cellular proteins binding Ca^2+^ are activated and trigger cellular processes. After the completion of signal transduction, the intracellular calcium concentration will quickly return to a low level. Therefore, the change of intracellular calcium concentration can indicate whether the cell is activated by some ligands. In a previous report, it had been proved that the activation of MRGPR receptors can cause the increase of intracellular calcium concentration [4]. In subsequent studies about MRGPR receptors, the change of intracellular calcium concentration was generally used to verify whether the MRGPR receptors were activated [1, 5-7]. Hence, we also use this same classical method to verify the activation of MRGPRX1 and other receptors by compound 16.

Secondly, the technology of calcium imaging was designed for detection of intracellular calcium concentration. A fluorescent dye (Fluo 4) that can bind calcium was used to realize the visualization and quantitative analysis of intracellular calcium. The maximum excitation and emission wavelength of Fluo 4 are 494 nm and 516 nm, respectively. The 488 nm wavelength light that can activate GFP commonly was used as the excitation of Fluo 4. When Fluo 4 was not combined with calcium, the fluorescence intensity is very weak under 488 nm excitation (Fig. 1A). However, when Fluo 4 was combined with calcium, the fluorescence intensity would increase rapidly (Fig. 1B). Thus, the level of intracellular calcium concentration can be indicated by the fluorescence intensity of Fluo 4. The two figures below showed the same cell field of vision before (A) and after (B) the treatment of compound 16, respectively.

Fig. 1 The fluorescence intensity changes of 293T cells transfected with MRGPRX1 before (A) and after (B) the treatment of compound 16 (500 nM) in the Fluo4-mediated calcium imaging experiment.

Thirdly, HEK293T cells transfected with plasmids (e.g. WT or point mutant MRGPRX1) were incubated with Fluo 4 dye for 40 min before the calcium imaging experiment. The fluorescence intensity of cells treated by Fluo 4 was detected under 488 nm excitation by ZEISS LSM880 confocal imaging system. The cells were treated with drugs (such as compound 16) between 80-100 seconds after the detection. The average fluorescence intensity of the cells before the drug treatment was named F0, and the real-time fluorescence intensity of the cells was named FT. FT/F0 was considered as the fluorescence intensity change of the cells between the drug treatment and non-treatment. The high value of FT/F0 represents the high intracellular calcium concentration, which suggested intense drug activation effect.

Hence, a trace represents the change of fluorescence intensity of a cell before and after drug treatment. The different traces represent different cells.

*3. Supplementary Figure 9: it is important to include positive controls, e.g. cognate agonists of each receptor, to show that the assays work for these receptors.*

**Response:** We thank the reviewer for the insightful advice. For calcium imaging assay, we used BAM8-22 (the well-studied agonist of MRGPRX1, 20 μM), C48/80 (the agonist of MRGPRX2, 20μg/ml) and DCA (Deoxycholic acid, the agonist of MRGPRX4, 20μM) as positive controls for MRGPRX1, MRGPRX2, and MRGPRX4. However, since there is no identified agonist of MRGPRX3 currently, we fail to find a positive control for MRGPRX3. Hence, we try to use 50 μM (a high enough concentration) compound 16 to treat MRGPRX3 and found no activated signals. Additionally, C48/80 and DCA are also used as positive controls in BRET assay to illustrate the weak agonist activity of compound 16 with MRGPRX2 and MRGPRX4. We believe these results could demonstrate that our assays work for these receptors. All these results are added in Supplementary Figure 13.

**Point-by-point response to Reviewer #2:**

***Reviewer #2 (Qing Fan, signs review):***

*The manuscript by B. Gan et al. describes the first cryo-EM structure of the itch receptor MRGPRX1 complexed with its cognate Gq protein. Although the structures of G protein-bound MRGPRX2 and MPGPRX4 receptors were reported in 2021, the MRGPRX1 structure reveals unique recognition pattern of a selective agonist, compound 16, within the orthosteric ligand-binding pocket. Compound 16 is a highly potent pain-relieving molecule that is specifically recognized by MRGPRX1 over opioid receptors. The molecular details of its interaction with the MRGPRX1 receptor that are provided in this study could facilitate the development of non-opioid drugs for pain. In addition, the authors performed functional analyses of the MRGPRX1 receptor using BRET and calcium imaging techniques as well as animal behavioral models. These functional data corroborated their structural findings on ligand binding specificity and receptor activation mechanism. The novelty and significance of this manuscript* *is sufficient for publishing in Plos Biology.* *Nevertheless, I have some concerns that need to be addressed before its publication.*

*1. The authors investigated the selectivity of Compound 16 for MRGPRX1 among various MRGPRXs. However, only a single dose of Compound 16 was used in Fig. S10 to determine the receptor specificity. Could the authors calculate the EC50 of compound 16 in MRGPRX1-4 activation using data from either the BRET or calcium mobilization assay? Can compound 16 activate MRGPRX2-4 receptors at a higher concentration than 500 nM, which was used to produce Fig. S10? These data may provide more rigorous proof for the receptor selectivity of compound 16.*

**Response:** We thank the reviewer for the constructive suggestion. We use BRET to evaluate the activation effect of compound 16 on MRGPRXs and generate four dose response curves (Fig. 2 A). Results show that MRGPRX2-4 cannot respond to compound 16. We calculate the EC_50_ of compound 16 in MRGPRX1 activation using data from BRET assay and calcium imaging assay (Fig. 2A and B). In addition, for calcium imaging assay, we further confirm that MRGPRX2-4 cannot be activated by compound 16 at the concentration of 50 μM (a high enough concentration) (Fig. 2C-E). These results are updated in Figure 4 and Supplementary Figure 13.

Fig. 2 Activation effect of compound 16 on MRGPRX1-X4. BRET assay result (A) and calcium imaging result (B). Representative calcium traces of MRGPRX2 (C), X3 (D) and X4 (E) responding to compound 16 (50μM). C48/80 (the agonist of MRGPRX2, 20μg/ml) and DCA (Deoxycholic acid, the agonist of MRGPRX4, 20μM) were used as positive controls.

*2. The residues at the binding interface of MRGPRX1 and Gαq need to be tested by mutagenesis and functional assays to validate the structural observations. The functional data may also be used to determine whether NanoBit tethering altered the binding pose of G protein to the receptor.*

**Response:** We thank the reviewer for the constructive suggestion. Alanine substitutions of residues at G_αq_ interface are tested using BERT assay, and the related results are updated in Supplementary Figure 9 and Supplementary Table 1. The detailed description has been added in our revised manuscript (Page 9, Line 14-26). We find that residues at ICL2 play more crucial roles in ligand-induced G_αq_ coupling than residues at TMs, which are in line with the results reported previously[8].

*3. Minor concerns*

*a. The size and labeling of various protein constructs in Fig. S2A need to be modified. Please double check the protein sizes of Gα and Gβ-HiBit to confirm their relative sizes and mobilities on SDS PAGE. In addition, the fusion construct should be Gβ-HiBit, not Gβγ-HiBit. Gβγ-HiBit would imply a covalently linked Gβ and Gγ construct that is further fused with HiBit.*

**Response:** We thank the reviewer for the careful inspection of our manuscript. After carefully check the construct sequences using in this study, we find that G_β_-HiBit with tag (42.0 KDa) is a bit larger than G_αq_ chimera (41.7 KDa). We apologize for our negligence and have corrected Supplementary Figure 2 in the revised manuscript.

*b. The authors appeared to have used a chimeric Gαq protein that was engineered based on a full-length Gs backbone and included mutations from a mini-Gαs/q construct. More information should be provided for this Gαq chimera in the main text, especially about its alpha helical domain (AHD), since this is different from the design of mini-G proteins.*

**Response:** We thank the reviewer for reviewing our manuscript carefully. The engineered G_αq_ chimera plasmid used in this study was donated by Prof. H. Eric Xu from Shanghai Institute of Materia Medica. The same Gαq chimera has been successfully used in the structure determination of several G_q_-bound GPCRs, including the G_q_-bound ghrelin receptor[9] and bradykinin receptors[10]. Here, we add a detailed description of the construct design in our revised main text (Page 4, Line 26-29; Page 5, Line 1-6) as well as methods (Page 15, Line 9-13), and provide an additional schematic diagram for clear presentation (Supplementary Figure 1D).

The modified text is attached below:

To further stabilize the complex, we used an engineered G_αq_ chimera in the complex assembly. The engineered G_αq_ chimera was designed based on the mini-G_αs/q71_[11, 12] with several modifications (Supplementary Figure 1D). Briefly, the N-terminal 1-18 residues of the mini-G_αs/q71_[11] were replaced by corresponding N-terminal sequences of the human G_αi1_, while the α-helical domain of G_αi1_ was subsequently inserted into the mini-G_αs/q71_, thus providing possible binding sites for two antibody fragments scFv16 and Fab-G50[13, 14]. Additionally, two dominant-negative mutations (G203A and A326S) were introduced to decrease the affinity of nucleotide binding[15]. The same engineered G_αq_ chimera had been successfully used in the structure determination of several G_q_-bound GPCRs, including the G_q_-bound ghrelin receptor[9] and bradykinin receptors[10] .

*c. Please provide a reference for the conserved P5.50I3.40F6.44 motif that was mentioned in the 'Activation of MRGPRX1' section.*

**Response:** We are sorry for the omission, and we have added the reference in our revised version (Page 9, Line 6).

**Point-by-point response to Reviewer #3:**

***Reviewer #3:***

*This manuscript reports a cryo-EM structure of the human mas-related G protein-coupled receptor X1 (MRGPRX1) bound to a synthetic agonist compound 16 and coupled to Gq protein. The structure revealed a unique pattern of residues in the orthosteric pocket that may facilitate the design of MRGPX1-selective compound. However, the authors apparently overlooked the manuscript by Liu et al published on October 27, 2022 in Nature Chemical Biology (*[*https://doi.org/10.1038/s41589-022-01173-6).*](https://doi.org/10.1038/s41589-022-01173-6).) *This manuscript describes an identical structure of the MRGPRX1 receptor in complex with compound 16 and Gq protein. As far as I can see, there are no new insights provided here compared to Liu et al, therefore I cannot recommend publication of this manuscript in its current form in PLOS Biology.*

**Response:** We appreciate for the time and efforts that the reviewer has taken to review our manuscript. We are sorry for overlooking the recently published manuscript. Liu et al[16] addressed some of the same issues and reported similar results as those we present in our manuscript. Here, we cite this related study and add a comparison of the recently reported structure contributed by Liu *et al*.[16] with our structure in the revised version. Results show that the two overall structures are similar, but the significant difference is the orientation of the phenylmethyl group in compound 16. Due to the better ligand density in our structure, compound 16 could be accommodated well with our proposed conformation. In contrast, the ligand leaves a part of the phenyl group out of the density map when we use Liu's structure to fit the density. Besides, in the updated manuscript, the results of conventional mouse models of itch demonstrate that compound 16 is a specific human MRGPRX1 agonist with high selectivity among species. Moreover, we also perform molecular docking to analyze the recognition difference between MRGPRX1 and MrgprA3 for CQ and propose that CQ weakly activates MRGPRX1 due to its weak interactions with critical hydrophobic residues in TM6.

**Point-by-point response to Reviewer #4:**

***Reviewer #4:***

*The data in this manuscript provide structural information on the human receptor MRGPRX1 in the presence of the agonist Compound 16. My task is to examine the manuscript in the context of pruritus. Why did you choose to use compound 16 as opposed to chloroquine? It is appreciated that chloroquine is not a potent agonist, but as it is considered by some investigators to be important in the context of MRGPRX1, it would be of interest to compare with compound 16, although it is recognized that this is a short report. Similarly, it would be of interest to have data using the mouse receptor MrgprA3.*

**Response:** We appreciate the reviewer for the insightful question and the constructive suggestion.

Actually, at the beginning of the study, we planned to solve the structure of MRGPRX1 in complex with CQ and compound 16. As two small molecule agonists of MRGPRX1, CQ shows extremely low potency (EC_50_: 297.68 μM) [1], while compound 16 exhibits high potency (EC_50_: 10.92 nM). We believed that solving the structure of MRGPRX1 bound to the two compounds with distinct binding affinity would help us understand the ligand recognition mechanism of MRGPRX1. However, we failed to obtain the stable CQ-bound MRGPRX1 complex with good protein behavior. Due to the difficulty and challenge in getting the GPCR in complex with weak agonist *in vitro*, more efforts should be made to complete this project in the future. Here, we performed molecular docking of MRGPRX1 with CQ to analyze the molecular basis of interactions between MRGPRX1 and CQ. The docking results will partially explain why CQ is a weak agonist of MRGPRX1 (Fig. 3A). Results show that CQ occupies a position away from TM6 in the binding pocket of MRGPRX1 compared with compound16 in the MRGPRX1-compound16 complex structure. It shows that the interactions between CQ and critical hydrophobic residues (F236^6.55^, F237^6.56^, and L240^6.59^) in TM6 are weaker than compound 16. Hence, we speculate that CQ weakly activates MRGPRX1 due to its weak interactions with critical hydrophobic residues in TM6.

Additionally, given that the potent compound 16 was initially designed to inhibit chronic pain by targeting MRGPRX1, we want to know the severity of itching that compound 16 might induce. To implement this idea, we compared the itch behavior of compound 16 with CQ in the conventional mouse model of itch. Our results show that compound 16 induces much less itch than a similar quantity of chloroquine. To further investigate why compound 16 behaves differently compared to CQ, we test the activation effects of compound 16 on MrgprA3 and MrgprC11. Our result shows that neither MrgprA3 nor MrgprC11 can be activated by compound 16 even at a concentration of 500 μM. Hence, we confirm that compound 16 is a specific human MRGPRX1 agonist with high selectivity among species.

Moreover, CQ shows higher potency to MrgprA3 (EC_50_: 27.55 μM) than MRGPRX1 (EC_50_: 297.68 μM)[1]. To understand the ligand recognition diversity of CQ among species, we built a structure model of MrgprA3 based on our MRGPRX1-compound 16 structure and performed molecular docking of CQ. The comparison results (Fig. 3B) show that the docking pose of CQ in MrgprA3 structure gets closer to TM6 when compared with results observed from Fig.3A. In MrgprA3, CQ occupies a position similar to the position compound 16 occupies in MRGPRX1, which are consistent with our previous speculations that stronger interactions with TM6 will improve the ligand binding affinity.

All these supplementary results are updated Figure 4 and Supplementary Figure 14. The related description is added in the discussion part (Page 11, Line 12-29; Page 12, Line 1-8). We believe our results can answer the question why CQ behaves differently in MRGPRX1 and MrgprA3.

Fig. 3 Comparisons of different ligand binding pockets from the magnified view. (A) A comparison between MRGPRX1-compound 16 and docking MRGPRX1 structure with CQ. (B) A comparison between MRGPRX1-compound 16 and docking MrgprA3 structure with CQ. Key residues and ligands are shown as sticks. MRGPRX1, docking MRGPRX1 and MrgprA3 are colored slate, palegreen, and gray, respectively. Compound 16 and CQ are colored cyan and hotpink, respectively.

*The last line of the Introduction should be toned down to: ...specifically and to potentially accelerate the development of a novel drug for the modulation of itch and pain.'*

**Response:** We apologize for the imprecise expression in our original manuscript and we have corrected this sentence as suggested (Page 4, Line 14-16).

*The second paragraph of the Discussion starts with 'Moreover, MRGPRX1 is reportedly....'. The second sentence beginning with Chloroquine needs to be modified. Chloroquine rarely causes itch, regardless of the references provided. In fact, hydroxychloroquine essentially never causes itch and is used widely for conditions other than malaria. I suggest: Chloroquine (CQ), a drug widely used in malaria treatment, can cause itch in some people.*

**Response:** We apologize for the imprecise expression of chloroquine and we thank the reviewer for the detailed comments on our manuscript. We have modified this sentence as suggested (Page 11, Line 2).

*Near the end of the Discussion, 'Sarah et al.' is noted. Please change Sarah to Wilson as the investigators name is Sarah Wilson.*

**Response:** We apologize for our negligence and we have corrected the citation as suggested (Page 12, Line 11-12).

*The sentence beginning with 'These data indicate' should be modified to 'These data are consistent with overlap between itch-sensing pathways and pain-sensing pathways.'*

**Response:** We apologize for the imprecise expression and we thank the reviewer for the careful inspection of our manuscript. We have modified the sentence as suggested. The modified sentence indeed improves the readability and totally convey what we want to express (Page 12, Line 14-15).

*In the Methods section, under Cryo-EM sample preparation, the beginning of the first sentence is awkward. I'm not sure what is meant by '3.5 µl of 12 mg/ml the concentrated complex'. Perhaps it simply missing an additional 'of' before 'the concentrated'.*

**Response:** We apologize for our grammatical errors in the original manuscript and we thank the reviewer for the detailed comments, and we have gone through the whole text to correct the similar grammar. This sentence has been corrected as “The concentrated sample (3.5 μL) at a concentration of 12 mg/mL was applied to glow-discharged holey carbon-coated grids (Quantifoil 200 mesh, Au R1.2/1.3).” (Page 17, Line 26-27).

Reference:

1.	Liu Q, Tang Z, Surdenikova L, Kim S, Patel KN, Kim A, et al. Sensory neuron-specific GPCR Mrgprs are itch receptors mediating chloroquine-induced pruritus. Cell. 2009;139(7):1353-65. Epub 2009/12/17. doi: 10.1016/j.cell.2009.11.034. PubMed PMID: 20004959; PubMed Central PMCID: PMCPMC2989405.

2.	Li Z, Tseng PY, Tiwari V, Xu Q, He SQ, Wang Y, et al. Targeting human Mas-related G protein-coupled receptor X1 to inhibit persistent pain. Proc Natl Acad Sci U S A. 2017;114(10):E1996-E2005. Epub 2017/02/23. doi: 10.1073/pnas.1615255114. PubMed PMID: 28223516; PubMed Central PMCID: PMCPMC5347560.

3.	Clapham DE. Calcium signaling. Cell. 2007;131(6):1047-58. Epub 2007/12/18. doi: 10.1016/j.cell.2007.11.028. PubMed PMID: 18083096.

4.	Han SK, Dong X, Hwang JI, Zylka MJ, Anderson DJ, Simon MI. Orphan G protein-coupled receptors MrgA1 and MrgC11 are distinctively activated by RF-amide-related peptides through the Galpha q/11 pathway. Proc Natl Acad Sci U S A. 2002;99(23):14740-5. doi: 10.1073/pnas.192565799. PubMed PMID: 12397184.

5.	McNeil BD, Pundir P, Meeker S, Han L, Undem BJ, Kulka M, et al. Identification of a mast-cell-specific receptor crucial for pseudo-allergic drug reactions. Nature. 2015;519(7542):237-41. Epub 2014/12/18. doi: 10.1038/nature14022. PubMed PMID: 25517090; PubMed Central PMCID: PMCPMC4359082.

6.	Meixiong J, Vasavda C, Green D, Zheng Q, Qi L, Kwatra SG, et al. Identification of a bilirubin receptor that may mediate a component of cholestatic itch. Elife. 2019;8. Epub 2019/01/19. doi: 10.7554/eLife.44116. PubMed PMID: 30657454; PubMed Central PMCID: PMCPMC6368403.

7.	Meixiong J, Vasavda C, Snyder SH, Dong X. MRGPRX4 is a G protein-coupled receptor activated by bile acids that may contribute to cholestatic pruritus. Proc Natl Acad Sci U S A. 2019;116(21):10525-30. Epub 2019/05/10. doi: 10.1073/pnas.1903316116. PubMed PMID: 31068464; PubMed Central PMCID: PMCPMC6535009.

8.	Cao C, Kang HJ, Singh I, Chen H, Zhang C, Ye W, et al. Structure, function and pharmacology of human itch GPCRs. Nature. 2021;600(7887):170-5. Epub 2021/11/19. doi: 10.1038/s41586-021-04126-6. PubMed PMID: 34789874.

9.	Wang Y, Guo S, Zhuang Y, Yun Y, Xu P, He X, et al. Molecular recognition of an acyl-peptide hormone and activation of ghrelin receptor. Nat Commun. 2021;12(1):5064. Epub 2021/08/22. doi: 10.1038/s41467-021-25364-2. PubMed PMID: 34417468; PubMed Central PMCID: PMCPMC8379176.

10.	Yin YL, Ye C, Zhou F, Wang J, Yang D, Yin W, et al. Molecular basis for kinin selectivity and activation of the human bradykinin receptors. Nat Struct Mol Biol. 2021;28(9):755-61. Epub 2021/09/15. doi: 10.1038/s41594-021-00645-y. PubMed PMID: 34518695.

11.	Nehme R, Carpenter B, Singhal A, Strege A, Edwards PC, White CF, et al. Mini-G proteins: Novel tools for studying GPCRs in their active conformation. PLoS One. 2017;12(4):e0175642. Epub 2017/04/21. doi: 10.1371/journal.pone.0175642. PubMed PMID: 28426733; PubMed Central PMCID: PMCPMC5398546.

12.	Wan Q, Okashah N, Inoue A, Nehmé R, Carpenter B, Tate CG, et al. Mini G protein probes for active G protein–coupled receptors (GPCRs) in live cells. Journal of Biological Chemistry. 2018;293(19):7466-73. doi: 10.1074/jbc.RA118.001975.

13.	Maeda S, Qu Q, Robertson MJ, Skiniotis G, Kobilka BKJS. Structures of the M1 and M2 muscarinic acetylcholine receptor/G-protein complexes. 2019;364(6440):552-7.

14.	Kang Y, Kuybeda O, de Waal PW, Mukherjee S, Van Eps N, Dutka P, et al. Cryo-EM structure of human rhodopsin bound to an inhibitory G protein. Nature. 2018;558(7711):553-8. Epub 2018/06/15. doi: 10.1038/s41586-018-0215-y. PubMed PMID: 29899450; PubMed Central PMCID: PMCPMC8054211.

15.	Liu P, Jia M-z, Zhou XE, De Waal PW, Dickson BM, Liu B, et al. The structural basis of the dominant negative phenotype of the Gαi1β1γ2 G203A/A326S heterotrimer. 2016;37(9):1259-72.

16.	Liu Y, Cao C, Huang XP, Gumpper RH, Rachman MM, Shih SL, et al. Ligand recognition and allosteric modulation of the human MRGPRX1 receptor. Nat Chem Biol. 2023;19(4):416-22. Epub 2022/10/28. doi: 10.1038/s41589-022-01173-6. PubMed PMID: 36302898.
