# Supplementary material for: Mechanism of agonist-induced activation of the human itch receptor MRGPRX1
Source: PLoS Biol. 2023 Jun 22;21(6):e3001975. doi: 10.1371/journal.pbio.3001975 (PMC10286997; doi:10.1371/journal.pbio.3001975)
Supplement: Peer review history

# Editor report: Decision Letter 0

**DOI:** 10.1371/journal.pbio.3001975.r001

**Authors:** Richard Hodge

19 Dec 2022

Dear Dr Ren, 

Thank you for submitting your manuscript entitled "The structural basis of itch receptor MRGPRX1 activation" for consideration as a Research Article by PLOS Biology. Again, I am very sorry for the delay in getting back to you as we consulted with an academic editor about your submission.

Your manuscript has now been evaluated by the PLOS Biology editorial staff, as well as by an academic editor with relevant expertise, and I am writing to let you know that we would like to send your submission out for external peer review.

However, before we can send your manuscript to reviewers, we need you to complete your submission by providing the metadata that is required for full assessment. To this end, please login to Editorial Manager where you will find the paper in the 'Submissions Needing Revisions' folder on your homepage. Please click 'Revise Submission' from the Action Links and complete all additional questions in the submission questionnaire.

Once your full submission is complete, your paper will undergo a series of checks in preparation for peer review. After your manuscript has passed the checks it will be sent out for review. To provide the metadata for your submission, please Login to Editorial Manager (https://www.editorialmanager.com/pbiology) within two working days, i.e. by Dec 21 2022 11:59PM.

If your manuscript has been previously peer-reviewed at another journal, PLOS Biology is willing to work with those reviews in order to avoid re-starting the process. Submission of the previous reviews is entirely optional and our ability to use them effectively will depend on the willingness of the previous journal to confirm the content of the reports and share the reviewer identities. Please note that we reserve the right to invite additional reviewers if we consider that additional/independent reviewers are needed, although we aim to avoid this as far as possible. In our experience, working with previous reviews does save time. 

If you would like us to consider previous reviewer reports, please edit your cover letter to let us know and include the name of the journal where the work was previously considered and the manuscript ID it was given. In addition, please upload a response to the reviews as a 'Prior Peer Review' file type, which should include the reports in full and a point-by-point reply detailing how you have or plan to address the reviewers' concerns. 

During the process of completing your manuscript submission, you will be invited to opt-in to posting your pre-review manuscript as a bioRxiv preprint. Visit http://journals.plos.org/plosbiology/s/preprints for full details. If you consent to posting your current manuscript as a preprint, please upload a single Preprint PDF. 

Feel free to email us at plosbiology@plos.org if you have any queries relating to your submission.

Kind regards,

Richard

Richard Hodge, PhD

Associate Editor, PLOS Biology

rhodge@plos.org

PLOS

Empowering researchers to transform science

Carlyle House, Carlyle Road, Cambridge, CB4 3DN, United Kingdom

ORCiD I plosbio.org I @PLOSBiology I Blog

California (U.S.) corporation #C2354500, based in San Francisco

# Peer review: Decision Letter 1

**DOI:** 10.1371/journal.pbio.3001975.r002

**Authors:** Richard Hodge

6 Feb 2023

Dear Dr Ren,

Thank you for your patience while your manuscript "The structural basis of itch receptor MRGPRX1 activation" was peer-reviewed at PLOS Biology. Please accept my apologies for the delays that you have experienced during the peer review process. It has now been evaluated by the PLOS Biology editors, an Academic Editor with relevant expertise, and by four independent reviewers. 

In light of the reviews, which you will find at the end of this email, we would like to invite you to revise the work to thoroughly address the reviewers' reports.

As you will see, the reviewers think the study is well done and provides novel insights into MRGPR pharmacology. However, they raise some overlapping concerns, including the lack of mutational validation to support the structure and binding mode of compound 16. In addition, Reviewer #3 raises concerns with the overall novelty of the manuscript since a recent study in Nature Chemical Biology has solved the the structure of MRGPRX1 in complex with compound 16 and Gq (https://doi.org/10.1038/s41589-022-01173-6). However, please note that the novelty of your study is protected under our scooping protection policy, given that the Nature Chemical Biology has been published within 6 months of your initial submission. We ask that you please cite this related study in the revised manuscript, as well as comparing the structures and placing your findings in context with the previous work. 

Given these reviews and after discussions with the academic editor, we also think that your manuscript would be a better fit as a Short Report at the journal. Upon resubmitting the revised manuscript, we ask that you please tick ‘Short Report’ as the article type and reduce to 4 main Figures. 

Given the extent of revision needed, we cannot make a decision about publication until we have seen the revised manuscript and your response to the reviewers' comments. Your revised manuscript is likely to be sent for further evaluation by all or a subset of the reviewers.

We expect to receive your revised manuscript within 3 months. Please email us (plosbiology@plos.org) if you have any questions or concerns, or would like to request an extension. 

At this stage, your manuscript remains formally under active consideration at our journal; please notify us by email if you do not intend to submit a revision so that we may withdraw it.

**IMPORTANT - SUBMITTING YOUR REVISION**

Your revisions should address the specific points made by each reviewer. Please submit the following files along with your revised manuscript:

1. A 'Response to Reviewers' file - this should detail your responses to the editorial requests, present a point-by-point response to all of the reviewers' comments, and indicate the changes made to the manuscript. 

*NOTE: In your point-by-point response to the reviewers, please provide the full context of each review. Do not selectively quote paragraphs or sentences to reply to. The entire set of reviewer comments should be present in full and each specific point should be responded to individually, point by point.

You should also cite any additional relevant literature that has been published since the original submission and mention any additional citations in your response. 

2. In addition to a clean copy of the manuscript, please also upload a 'track-changes' version of your manuscript that specifies the edits made. This should be uploaded as a "Revised Article with Changes Highlighted" file type. 

*Re-submission Checklist*

When you are ready to resubmit your revised manuscript, please refer to this re-submission checklist: https://plos.io/Biology_Checklist

To submit a revised version of your manuscript, please go to https://www.editorialmanager.com/pbiology/ and log in as an Author. Click the link labelled 'Submissions Needing Revision' where you will find your submission record. 

Please make sure to read the following important policies and guidelines while preparing your revision:

*Published Peer Review*

Please note while forming your response, if your article is accepted, you may have the opportunity to make the peer review history publicly available. The record will include editor decision letters (with reviews) and your responses to reviewer comments. If eligible, we will contact you to opt in or out. Please see here for more details:

https://blogs.plos.org/plos/2019/05/plos-journals-now-open-for-published-peer-review/

*PLOS Data Policy*

Please note that as a condition of publication PLOS' data policy (http://journals.plos.org/plosbiology/s/data-availability) requires that you make available all data used to draw the conclusions arrived at in your manuscript. If you have not already done so, you must include any data used in your manuscript either in appropriate repositories, within the body of the manuscript, or as supporting information (N.B. this includes any numerical values that were used to generate graphs, histograms etc.). For an example see here: http://www.plosbiology.org/article/info%3Adoi%2F10.1371%2Fjournal.pbio.1001908#s5

*Blot and Gel Data Policy*

We require the original, uncropped and minimally adjusted images supporting all blot and gel results reported in an article's figures or Supporting Information files. We will require these files before a manuscript can be accepted so please prepare them now, if you have not already uploaded them. Please carefully read our guidelines for how to prepare and upload this data: https://journals.plos.org/plosbiology/s/figures#loc-blot-and-gel-reporting-requirements

*Protocols deposition*

To enhance the reproducibility of your results, we recommend that if applicable you deposit your laboratory protocols in protocols.io, where a protocol can be assigned its own identifier (DOI) such that it can be cited independently in the future. Additionally, PLOS ONE offers an option for publishing peer-reviewed Lab Protocol articles, which describe protocols hosted on protocols.io. Read more information on sharing protocols at https://plos.org/protocols?utm_medium=editorial-email&utm_source=authorletters&utm_campaign=protocols

Thank you again for your submission to our journal. We hope that our editorial process has been constructive thus far, and we welcome your feedback at any time. Please don't hesitate to contact us if you have any questions or comments.

Sincerely,

Richard

Richard Hodge, PhD

Associate Editor, PLOS Biology

rhodge@plos.org

------------------------------------

REVIEWS:

Reviewer #1: This manuscript from Gan etc. reports a cryo-EM structure of the MRGPRX1-Gq complex with compound 16. The structure revealed a novel binding pose of compound 16 and a unique agonist recognition pattern compared to other MRGPRXs. Interestingly, the authors showed that compound 16 induced a much lowered itchy response compared to another MRGPRX1 agonist in an animal assay. The overall structural determination work is fine. The cryo-EM density map seems to allow unambiguous modeling. The results provide novel insight into MRGPR pharmacology.

One major issue is the lack of mutagenesis studies to support their structural findings. Considering the novel binding pose of compound 16, adequate mutagenesis studies are critical. Another major issue is the very limited discussion of their functional assay. Why compound 16 behaved differently compared to CQ? Do they induce different signaling pathways downstream of MRGPRX1? 

Other minor issues:

1. It would be informative to discuss the potential mechanism by which compound 16 activates MRGPRX1. The authors only discussed the changes of some conserved microdomains in MRGPRX1.

2. Figure 5A: What are the different traces? Also, does this figure just tell us compound 16 is a MRGPRX1 agonist? Any new information from this figure?

3. Supplementary Figure 9: it is important to include positive controls, e.g. cognate agonists of each receptor, to show that the assays work for these receptors. 

Reviewer #2 (Qing Fan, signs review): The manuscript by B. Gan et al. describes the first cryo-EM structure of the itch receptor MRGPRX1 complexed with its cognate Gq protein. Although the structures of G protein-bound MRGPRX2 and MPGPRX4 receptors were reported in 2021, the MRGPRX1 structure reveals unique recognition pattern of a selective agonist, compound 16, within the orthosteric ligand-binding pocket. Compound 16 is a highly potent pain-relieving molecule that is specifically recognized by MRGPRX1 over opioid receptors. The molecular details of its interaction with the MRGPRX1 receptor that are provided in this study could facilitate the development of non-opioid drugs for pain. In addition, the authors performed functional analyses of the MRGPRX1 receptor using BRET and calcium imaging techniques as well as animal behavioral models. These functional data corroborated their structural findings on ligand binding specificity and receptor activation mechanism. The novelty and significance of this manuscript is sufficient for publishing in Plos Biology. Nevertheless, I have some concerns that need to be addressed before its publication.

1. The authors investigated the selectivity of Compound 16 for MRGPRX1 among various MRGPRXs. However, only a single dose of Compound 16 was used in Fig. S10 to determine the receptor specificity. Could the authors calculate the EC50 of compound 16 in MRGPRX1-4 activation using data from either the BRET or calcium mobilization assay? Can compound 16 activate MRGPRX2-4 receptors at a higher concentration than 500 nM, which was used to produce Fig. S10? These data may provide more rigorous proof for the receptor selectivity of compound 16.

2. The residues at the binding interface of MRGPRX1 and Gαq need to be tested by mutagenesis and functional assays to validate the structural observations. The functional data may also be used to determine whether NanoBit tethering altered the binding pose of G protein to the receptor.

3. Minor concerns

a. The size and labeling of various protein constructs in Fig. S2A need to be modified. Please double check the protein sizes of Gα and Gβ-HiBit to confirm their relative sizes and mobilities on SDS PAGE. In addition, the fusion construct should be Gβ-HiBit, not Gβγ-HiBit. Gβγ-HiBit would imply a covalently linked Gβ and Gγ construct that is further fused with HiBit.

b. The authors appeared to have used a chimeric Gαq protein that was engineered based on a full-length Gs backbone and included mutations from a mini-Gαs/q construct. More information should be provided for this Gαq chimera in the main text, especially about its alpha helical domain (AHD), since this is different from the design of mini-G proteins. 

c. Please provide a reference for the conserved P5.50I3.40F6.44 motif that was mentioned in the 'Activation of MRGPRX1' section. 

Reviewer #3: This manuscript reports a cryo-EM structure of the human mas-related G protein-coupled receptor X1 (MRGPRX1) bound to a synthetic agonist compound 16 and coupled to Gq protein. The structure revealed a unique pattern of residues in the orthosteric pocket that may facilitate the design of MRGPX1-selective compound. However, the authors apparently overlooked the manuscript by Liu et al published on October 27, 2022 in Nature Chemical Biology (https://doi.org/10.1038/s41589-022-01173-6). This manuscript describes an identical structure of the MRGPRX1 receptor in complex with compound 16 and Gq protein. As far as I can see, there are no new insights provided here compared to Liu et al, therefore I cannot recommend publication of this manuscript in its current form in PLOS Biology.

Reviewer #4: The data in this manuscript provide structural information on the human receptor MRGPRX1 in the presence of the agonist Compound 16. My task is to examine the manuscript in the context of pruritus. Why did you choose to use compound 16 as opposed to chloroquine? It is appreciated that chloroquine is not a potent agonist, but as it is considered by some investigators to be important in the context of MRGPRX1, it would be of interest to compare with compound 16, although it is recognized that this is a short report. Similarly, it would be of interest to have data using the mouse receptor MrgprA3. The last line of the Introduction should be toned down to: ...specifically and to potentially accelerate the development of a novel drug for the modulation of itch and pain.'

The second paragraph of the Discussion starts with 'Moreover, MRGPRX1 is reportedly....'. The second sentence beginning with Chloroquine needs to be modified. Chloroquine rarely causes itch, regardless of the references provided. In fact, hydroxychloroquine essentially never causes itch and is used widely for conditions other than malaria. I suggest: Chloroquine (CQ), a drug widely used in malaria treatment, can cause itch in some people.

Near the end of the Discussion, 'Sarah et al.' is noted. Please change Sarah to Wilson as the investigators name is Sarah Wilson.

The sentence beginning with 'These data indicate' should be modified to 'These data are consistent with overlap between itch-sensing pathways and pain-sensing pathways.'

In the Methods section, under Cryo-EM sample preparation, the beginning of the first sentence is awkward. I'm not sure what is meant by '3.5 µl of 12 mg/ml the concentrated complex'. Perhaps it simply missing an additional 'of' before 'the concentrated'.

# Author comment: Author response to Decision Letter 1

**DOI:** 10.1371/journal.pbio.3001975.r003

3 May 2023

Attachment Submitted filename: R_20230428_Response to reviewers.docx Click here for additional data file.

# Editor report: Decision Letter 2

**DOI:** 10.1371/journal.pbio.3001975.r004

**Authors:** Richard Hodge

11 May 2023

Dear Dr Ren,

Thank you for your patience while we considered your revised manuscript "The structural basis of itch receptor MRGPRX1 activation" for publication as a Short Report at PLOS Biology. This revised version of your manuscript has been evaluated by the PLOS Biology editors and the Academic Editor.

Based on our Academic Editor's assessment of your revision, I am pleased to say that we are likely to accept this manuscript for publication, provided you satisfactorily address the following data and other policy-related requests that I have provided below (A-H):

(A) We would like to suggest the following modification to the title: 

"Mechanism of agonist-induced activation of the human itch receptor MRGPRX1"

(B) In the animal ethics statement in the Methods section of the manuscript, please specifically state that your IACUC approved your animal studies. In addition, please provide the method of euthanasia used to sacrifice the mice.

(C) You may be aware of the PLOS Data Policy, which requires that all data be made available without restriction: http://journals.plos.org/plosbiology/s/data-availability. For more information, please also see this editorial: http://dx.doi.org/10.1371/journal.pbio.1001797

Note that we do not require all raw data. Rather, we ask that all individual quantitative observations that underlie the data summarized in the figures and results of your paper be made available in one of the following forms:

-Supplementary files (e.g., excel). Please ensure that all data files are uploaded as 'Supporting Information' and are invariably referred to (in the manuscript, figure legends, and the Description field when uploading your files) using the following format verbatim: S1 Data, S2 Data, etc. Multiple panels of a single or even several figures can be included as multiple sheets in one excel file that is saved using exactly the following convention: S1_Data.xlsx (using an underscore).

-Deposition in a publicly available repository. Please also provide the accession code or a reviewer link so that we may view your data before publication. 

Regardless of the method selected, please ensure that you provide the individual numerical values that underlie the summary data displayed in the following figure panels as they are essential for readers to assess your analysis and to reproduce it:

Figure 2D, 2G-H, 3D, 4A-D, S1C, S9C, S9E-F, S13I-M, S15A-C

NOTE: the numerical data provided should include all replicates AND the way in which the plotted mean and errors were derived (it should not present only the mean/average values).

(D) Thank you for depositing the structural data in the PDB (8HJ5) and EMDB (EMD-34833) databases. However, I note that the data is currently on hold. We ask that you please make this data publicly available before publication.

(E) Please also ensure that each of the relevant figure legends in your manuscript include information on *WHERE THE UNDERLYING DATA CAN BE FOUND*, and ensure your supplemental data file/s has a legend.

(F) We require the original, uncropped and minimally adjusted images supporting all blot and gel results reported in the following Figures:

Figure S2A

We will require these files before a manuscript can be accepted so please prepare and upload them now. Please carefully read our guidelines for how to prepare and upload this data: https://journals.plos.org/plosbiology/s/figures#loc-blot-and-gel-reporting-requirements

(H) Please ensure that your Data Statement in the submission system accurately describes where your data can be found and is in final format, as it will be published as written there. 

------------------------------------

As you address these items, please take this last chance to review your reference list to ensure that it is complete and correct. If you have cited papers that have been retracted, please include the rationale for doing so in the manuscript text, or remove these references and replace them with relevant current references. Any changes to the reference list should be mentioned in the cover letter that accompanies your revised manuscript.

We expect to receive your revised manuscript within two weeks. 

To submit your revision, please go to https://www.editorialmanager.com/pbiology/ and log in as an Author. Click the link labelled 'Submissions Needing Revision' to find your submission record. Your revised submission must include the following:

- a cover letter that should detail your responses to any editorial requests, if applicable, and whether changes have been made to the reference list

- a Response to Reviewers file that provides a detailed response to the reviewers' comments (if applicable)

- a track-changes file indicating any changes that you have made to the manuscript. 

NOTE: If Supporting Information files are included with your article, note that these are not copyedited and will be published as they are submitted. Please ensure that these files are legible and of high quality (at least 300 dpi) in an easily accessible file format. For this reason, please be aware that any references listed in an SI file will not be indexed. For more information, see our Supporting Information guidelines:

https://journals.plos.org/plosbiology/s/supporting-information

*Published Peer Review History*

Please note that you may have the opportunity to make the peer review history publicly available. The record will include editor decision letters (with reviews) and your responses to reviewer comments. If eligible, we will contact you to opt in or out. Please see here for more details:

https://blogs.plos.org/plos/2019/05/plos-journals-now-open-for-published-peer-review/

*Press*

Should you, your institution's press office or the journal office choose to press release your paper, please ensure you have opted out of Early Article Posting on the submission form. We ask that you notify us as soon as possible if you or your institution is planning to press release the article.

*Protocols deposition*

To enhance the reproducibility of your results, we recommend that if applicable you deposit your laboratory protocols in protocols.io, where a protocol can be assigned its own identifier (DOI) such that it can be cited independently in the future. Additionally, PLOS ONE offers an option for publishing peer-reviewed Lab Protocol articles, which describe protocols hosted on protocols.io. Read more information on sharing protocols at https://plos.org/protocols?utm_medium=editorial-email&utm_source=authorletters&utm_campaign=protocols

Please do not hesitate to contact me should you have any questions.

Kind regards,

Richard

Richard Hodge, PhD

Associate Editor, PLOS Biology

rhodge@plos.org

PLOS

Empowering researchers to transform science

Carlyle House, Carlyle Road, Cambridge, CB4 3DN, United Kingdom

ORCiD I plosbio.org I @PLOSBiology I Blog

California (U.S.) corporation #C2354500, based in San Francisco

# Author comment: Author response to Decision Letter 2

**DOI:** 10.1371/journal.pbio.3001975.r005

24 May 2023

Attachment Submitted filename: R_20230428_Response to reviewers.docx Click here for additional data file.

# Editor report: Decision Letter 3

**DOI:** 10.1371/journal.pbio.3001975.r006

**Authors:** Richard Hodge

31 May 2023

Dear Dr Ren,

Thank you for the submission of your revised Short Report "Mechanism of agonist-induced activation of the human itch receptor MRGPRX1" for publication in PLOS Biology. On behalf of my colleagues and the Academic Editor, Raimund Dutzler, I am pleased to say that we can accept your manuscript for publication, provided you address any remaining formatting and reporting issues. These will be detailed in an email you should receive within 2-3 business days from our colleagues in the journal operations team; no action is required from you until then. Please note that we will not be able to formally accept your manuscript and schedule it for publication until you have completed any requested changes.

Please take a minute to log into Editorial Manager at http://www.editorialmanager.com/pbiology/, click the "Update My Information" link at the top of the page, and update your user information to ensure an efficient production process.

PRESS

We frequently collaborate with press offices. If your institution or institutions have a press office, please notify them about your upcoming paper at this point, to enable them to help maximise its impact. If the press office is planning to promote your findings, we would be grateful if they could coordinate with biologypress@plos.org. If you have previously opted in to the early version process, we ask that you notify us immediately of any press plans so that we may opt out on your behalf.

We also ask that you take this opportunity to read our Embargo Policy regarding the discussion, promotion and media coverage of work that is yet to be published by PLOS. As your manuscript is not yet published, it is bound by the conditions of our Embargo Policy. Please be aware that this policy is in place both to ensure that any press coverage of your article is fully substantiated and to provide a direct link between such coverage and the published work. For full details of our Embargo Policy, please visit http://www.plos.org/about/media-inquiries/embargo-policy/.

Thank you again for choosing PLOS Biology for publication and supporting Open Access publishing. We look forward to publishing your study. 

Best wishes, 

Richard

Richard Hodge, PhD

Associate Editor, PLOS Biology

rhodge@plos.org

PLOS

Empowering researchers to transform science

Carlyle House, Carlyle Road, Cambridge, CB4 3DN, United Kingdom

ORCiD I plosbio.org I @PLOSBiology I Blog

California (U.S.) corporation #C2354500, based in San Francisco
